# High-throughput screening against protein:protein interaction interfaces reveals anti-cancer therapeutics as potent modulators of the voltage-gated Na^+^ channel complex

**DOI:** 10.1038/s41598-019-53110-8

**Published:** 2019-11-15

**Authors:** Paul A. Wadsworth, Oluwarotimi Folorunso, Nghi Nguyen, Aditya K. Singh, Daniela D’Amico, Reid T. Powell, David Brunell, John Allen, Clifford Stephan, Fernanda Laezza

**Affiliations:** 1 MD/PhD Combined Degree Program and Biochemistry and Molecular Biology Graduate Program, The University of Texas Medical Branch, Galveston, Texas 77555 USA; 2 Department of Pharmacology & Toxicology, The University of Texas Medical Branch, Galveston, Texas 77555 USA; 3 HTS Screening Core, Center for Translational Cancer Research, Texas A&M Health Science Center: Institute of Biosciences and Technology, Houston, TX 77030 USA; 4 Neuroscience Graduate Program, The University of Texas Medical Branch, Galveston, Texas 77555 USA

**Keywords:** Sodium channels, Pharmacology, High-throughput screening, Target identification

## Abstract

Multiple voltage-gated Na^+^ (Nav) channelopathies can be ascribed to subtle changes in the Nav macromolecular complex. Fibroblast growth factor 14 (FGF14) is a functionally relevant component of the Nav1.6 channel complex, a causative link to spinocerebellar ataxia 27 (SCA27) and an emerging risk factor for neuropsychiatric disorders. Yet, how this protein:channel complex is regulated in the cell is still poorly understood. To search for key cellular pathways upstream of the FGF14:Nav1.6 complex, we have developed, miniaturized and optimized an in-cell assay in 384-well plates by stably reconstituting the FGF14:Nav1.6 complex using the split-luciferase complementation assay. We then conducted a high-throughput screening (HTS) of 267 FDA-approved compounds targeting known mediators of cellular signaling. Of the 65 hits initially detected, 24 were excluded based on counter-screening and cellular toxicity. Based on target analysis, potency and dose-response relationships, 5 compounds were subsequently repurchased for validation and confirmed as hits. Among those, the tyrosine kinase inhibitor lestaurtinib was highest ranked, exhibiting submicromolar inhibition of FGF14:Nav1.6 assembly. While providing evidence for a robust in-cell HTS platform that can be adapted to search for any channelopathy-associated regulatory proteins, these results lay the potential groundwork for repurposing cancer drugs for neuropsychopharmacology.

## Introduction

Voltage-gated sodium (Nav) channels are the molecular determinant of the action potential, which underlies electrical signaling in the brain. Protein:protein interactions (PPI) between Nav channels and their accessory proteins fine-tune neuronal excitability, and mutations in either the channel itself^1,2^ or these regulatory proteins^3–8^ give rise to channelopathies that have few viable treatment options. PPI interfaces are specific and flexible, making them ideal scaffolds for probe and drug design^9,10^, especially within the CNS where selectivity and specificity are vital for limiting side effects^11^.

Studies have shown that the PPI complex between the Nav1.6 channel and its accessory regulator protein, fibroblast growth factor 14 (FGF14), is a functionally relevant regulator of neuronal excitability in the cortico-mesolimbic circuit and cerebellum^12–24^. Single-nucleotide polymorphisms in exonic regions of *FGF14* cause spinocerebellar ataxia 27 (SCA27), an autosomal dominant disease associated with complex neuropsychiatric symptoms^6–8,12,21,25,26^, while intronic SNPs or changes in the expression level of FGF14 have been linked to schizophrenia and other neuropsychiatric disorders^4,5,17^. FGF14 binds to the Nav1.6 intracellular C-terminal domain and promotes localization of Nav1.6 channels to the proximal region of the axon, which is the primary initiation site of the action potential^12,14,20,23,27–31^. Interactions between FGF14 and Nav1.6 are regulated by kinase signaling pathways including glycogen synthase kinase 3 (GSK3) and casein kinase 2 (CK2), which directly phosphorylate serine/threonine (S/T) sites on FGF14 and/or Nav1.6. Targeting these kinases with inhibitors or short-hairpin RNA alters protein complex stability, Nav1.6 currents and excitability^13,16,19,27,32^, while peptidomimetics targeting the FGF14^V160^ and FGF14^Y158^ residues, which are located at the FGF14:Nav1.6 PPI interface, reduce complex formation, exhibit state-dependent modulation of Nav1.6 currents and suppress excitability of medium spiny neurons in the nucleus accumbens (NAc)^20,28^. These findings not only provide evidence for druggability of the FGF14:Nav1.6 complex but also suggest that modulation of cell signaling could provide a strategy for rescuing function of the Nav1.6 channel or FGF14 in related channelopathies.

**Figure 1:**
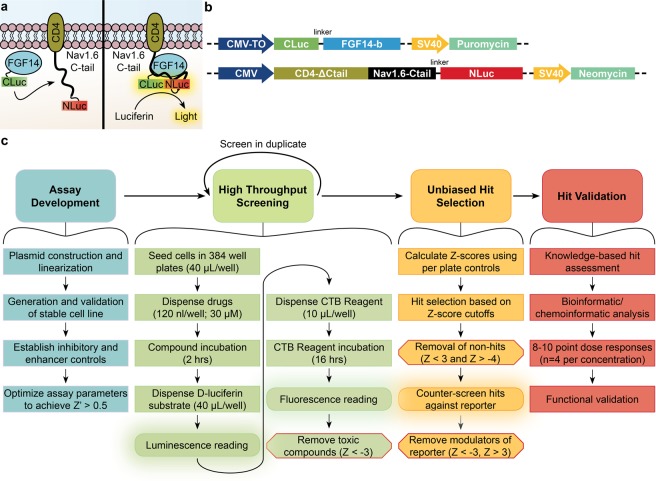
Overview of the cell-based LCA for HTS against the FGF14:Nav1.6 C-tail complex. (**a**) Theory of LCA in live cells. Assembly of the CLuc-FGF14:CD4-Nav1.6-NLuc complex results in reconstitution of the luciferase enzymatic activity, which produces light in the presence of its substrate D-luciferin. (**b**) Linearized constructs encoding CLuc-FGF14-1b and CD4-Nav1.6-NLuc under the control of Neomycin and Puromycin, respectively, were sequentially transfected into HEK293 cells to create the double stable cell line. (**c**) Workflow for HTS using a cell-based assay. The work presented here describes assay development, screening and counter-screening of a test library of kinase inhibitors, and preliminary dose response hit validation. Z′ was used to measure the assay’s ability to detect hits, whereas Z-scores were calculated for experimental compounds based on per plate controls. CTB, CellTiter Blue (cell viability assay).

Identifying new modulators of PPI within ion channel complexes has been hampered by the lack of robust in-cell assays and screening platforms. To address this need, we describe the development and optimization of an in-cell split luciferase complementation assay (LCA) suitable for screening changes in PPI between FGF14 and its associated Nav1.6 interacting domain, the intracellular C-terminal tail of the channel, in 384-well plates (Fig. 1). Further, we present the screening results from the Custom Clinical and National Cancer Institute (CC_NCI) collection of 267 FDA-approved drugs targeting known cellular signaling pathways, which was used as a test library for our assay. Our study not only provides a new practical tool to accelerate drug discovery for ion channels, but also identifies the tyrosine kinase inhibitor lestaurtinib, an FDA approved anti-cancer drug, as a potential compound for repurposing toward CNS-related channelopathies.

## Results

### Development of a robust assay to assess FGF14:Nav1.6 C-tail interactions in a double stable HEK293 cell line

We have previously introduced the LCA to detect interactions between FGF14 and the Nav1.6 C-tail in transiently transfected cells^15,16,19^. The C- and N-terminal fragments of the *P. Pyralis* luciferase are fused, respectively, to FGF14 (CLuc-FGF14) and a chimera expressing CD4 fused to the Nav1.6 C-tail (CD4-Nav1.6-NLuc), and FGF14:Nav1.6 C-tail complex formation can be detected in the presence of the luciferase substrate, D-luciferin (Fig. 1a–c). In order to utilize this system for HTS, we developed a double stable cell line that increased signal-to-noise ratio, decreased well-to-well variability, and circumvented the need for high volume transient transfections, which are labor-intensive and uneconomical. We generated a monoclonal double stable cell line by sequentially transfecting HEK293 cells with linearized CLuc-FGF14 and CD4-Nav1.6C-tail-NLuc constructs (Fig. 1b) under the control of puromycin and neomycin, respectively.

**Figure 2:**
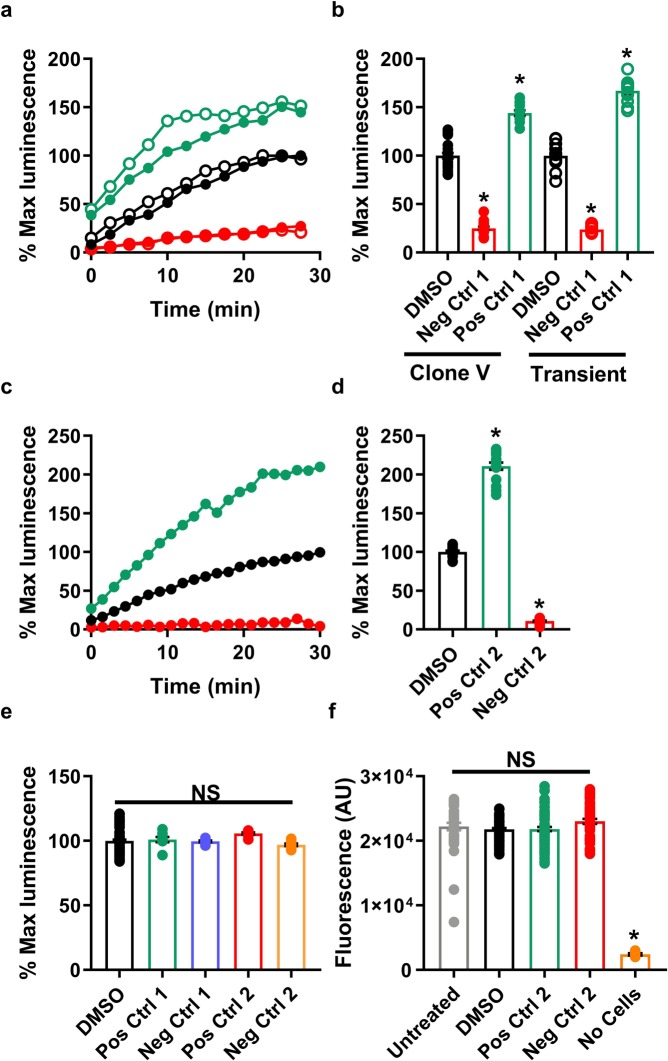
Validation of double stable cell line and selection of inhibitory and enhancer controls. (**a,b**) Clone V cells (filled circles) stably expressing CLuc-FGF14 and CD4-Nav1.6-C-tail-NLuc constructs were compared with transiently transfected HEK293 cells (empty circles) treated with the Akt inhibitor triciribine (positive control 1, green) or the peptidomimetic ZL181 (negative control 1, red). (**a**) Percent luminescence (normalized to 0.3% DMSO controls, *n* = 16) is measured over 30 minutes following dispensing of luciferin substrate in 96-well plates, and (**b**) percent of maximal luminescence for each group shown in (**a**). (**c**,**d**) TNF-α (positive control 2, green) and MNS (negative control 2, red) are more potent than original positive and negative controls (triciribine and ZL181 in (**a**,**b**)) and demonstrate the high performance capabilities of LCA as an HTS assay. (**c**) Percent luminescence over time and (**d**) percent maximal luminescence of Clone V cells after treatment with 50 ng/mL TNF-α (positive control 2, green) or 25 µM MNS (negative control 2, red) in 96-well plates. (**e**) HEK293 cells were transiently transfected with full-length *P. pyralis* luciferase to rule out effects on luciferase following treatment with triciribine (positive control 1, green), ZL181 (negative control 1, purple), 50 ng/mL TNF-α (positive control 2, red) or 30 µM MNS (negative control 2, orange) in 96-well plates. Percent maximal luminescence (normalized to 0.3% DMSO controls, *n* = 16) is shown for each treatment (*n* = 8 per treatment), and no significant effects were observed. **(f)** Cell titer blue (CTB) assay was initiated on Clone V cells or wells containing media alone immediately following luminescence reading in 384-well plates (*n = *16 per group) by dispensing 30 µL of CTB reagent. Fluorescence intensities were subsequently read after approximately 18 hours. Untreated cells (white), 0.3% DMSO (gray), 25 µM MNS (red), 50 ng/mL TNF-α (green), or media and luciferin mixture with no cells (orange). Data are mean for real-time graphs (**a**,**c**) and values measured from individual replicate wells are plotted for graphs showing % maximal luminescence (B,D,E) or fluorescence (**f**). One-way ANOVA with post-hoc Dunnett’s multiple comparisons test was used to determine significance; *p < 0.0001.

Next, we compared the double stable cell line, hereafter referred to as Clone V, to transiently transfected HEK293 cells after treatment with the peptidomimetic ZL181 (negative control 1), a rationally designed inhibitor of the FGF14:Nav1.6 interaction^28^, and the Akt inhibitor triciribine (positive control 1), which enhances the interaction of this complex presumably by increasing GSK3-dependent phosphorylation of the complex^19,32^. Treatment with 50 µM ZL181 caused a similar inhibitory effect (23.9 vs. 25% luminescence compared to DMSO control), and treatment with 25 µM triciribine resulted in a similar increase in FGF14:Nav1.6 C-tail assembly (144.0% vs. 166.9% luminescence) in transiently transfected cells compared to Clone V cells (Fig. 2a,b). At this stage, our use of ZL181 and triciribine as controls was to validate that Clone V behaved similar to transiently transfected cells as shown previously^19,28,32^. While an enhancer acting through more direct means may be preferable, this limitation arises from the very problem that this HTS project aims to solve; namely, to discover specific and potent modulators of the FGF14:Nav1.6 complex.

### Selection of potent inhibitory and enhancer controls suitable for an HTS format

Following validation of Clone V, we scaled our assay from a 96-well to 384-well plate format to economically support high-throughput drug screening. We chose conditions leading to satisfactory assay performance that minimally impacted assay sensitivity, and we calculated Z′-factor (Eq. 1) to evaluate the robustness of our assay. Z′-factor measures the signal separation between the mean of positive and negative controls and the variance between replicates. To improve Z′-factor, the inhibitory (negative) control should reduce the signal to as close to zero percent as possible, while an enhancer (positive) control should increase the signal by ≥2-fold and ≥3 SDs (i.e., ≥200% luminescence when normalized to DMSO controls). However, ZL181 plateaus at ~25% luminescence^28^, while the enhancing effect of triciribine plateaus at ~150% luminescence^19^. Thus, we searched for controls with greater potency and efficacy than that of ZL181 and triciribine.

Parallel studies lead us to explore the effect of TNF-α (positive control 2) on the FGF14:Nav1.6 complex, and we found that it is a more efficacious enhancer of the complex (mean and SD: TNF-α, 210.8% ± 18.6%; triciribine, 142.5% ± 10.7%; triciribine, 142.5% ± 10.7%; Fig. 2a–d); despite moderately higher variance, the mean effect of TNF-α is over 2-fold greater than that of triciribine. Additionally, we found that the tyrosine kinase inhibitor MNS (30 µM; negative control 2) significantly reduces FGF14:Nav1.6 interaction, and the effect was greater with lower variance than that of ZL181 (one-way ANOVA; normalized mean and SD: MNS, 10.8% ± 3.1%; ZL181, 25.0% ± 7.3%; p < 0.0001) (Fig. 2).

The concentration range of MNS and TNF-α was selected based on a preliminary dose response (Supplementary Fig. S1). TNF-α protein supplemented with 0.1 mg/mL BSA (manufacturer suggestion) improves protein stability, increases efficacy, and minimizes variance (Supplementary Fig. S1). However, the increased viscosity of this solution was problematic for dispensing using the LabCyte Echo 550. Thus, using a higher concentration of TNF-α without BSA (50 ng/mL) reproduced the effects observed for lower TNF-α concentrations (5 and 25 ng/mL) supplemented with BSA.

These new positive and negative controls were subsequently validated to ensure that their respective effects on luminescence arose due to modulation of PPI between FGF14 and the Nav1.6 C-tail rather than confounding factors. To rule out that the observed luminescence change by these compounds was a result of interference with luciferase enzymatic activity, a common side-effect in luminescence-based assays, HEK293 cells transfected with the full-length *P. pyralis* luciferase were similarly treated with MNS (30 µM) or TNF-α (50 ng/mL) in 96-well plates (DMSO, *n* = 32; MNS and TNF-α, *n* = 4 per group). There was no significant effect observed (one-way ANOVA; normalized mean and SD: DMSO, 100 ± 10.3%; triciribine, 101.0 ± 5.8%; ZL181, 99.58 ± 1.8%; MNS, 96.8% ± 2.8%; TNF-α, 105.7% ± 2.5%; Fig. 2f). Next, we used western blot to confirm that Clone V cells expressed TNF receptor 1 (TNFR1), the primary receptor that initiates TNF-α signaling cascades^33,34^ (Supplementary Fig. S1). Additionally, we used western blot to rule out changes in expression of recombinant CLuc-FGF14 or CD4-Nav1.6-NLuc protein as a potential mediator of changes in luminescence from Clone V cells treated with MNS or TNF-α (Supplementary Fig. S1). Finally, the CellTiter Blue (CTB) cell viability assay was used as a counter screen to eliminate drug toxicity as a confounding variable for luminescence signal intensity. The CTB reagent was dispensed into 384-wells immediately after LCA luminescence reading, and fluorescence was read after 16 hrs. We observed no significant difference in cell viability between untreated cells (media alone) or cells treated with 0.3% DMSO, 25 µM MNS, or 50 ng/mL TNF-α (Fig. 2). These new control compounds demonstrate that our modified LCA is capable of detecting agents that greatly increase or decrease FGF14:Nav1.6 complex formation without modifying the assay output (luminescence) through non-specific effects (i.e., luciferase modulation or changes in protein expression).

### Optimization of assay parameters in 384-well plates

These controls were subsequently used as guides as we miniaturized the assay format from 96-well to 384-well plates. We first optimized cell plating time and media composition, and subsequently used these conditions to explore the effects of cell density and substrate incubation times on assay sensitivity (Z′-factor).

Previously, transiently transfected cells were plated 24 hrs prior to reading in order to facilitate protein production and cell adhesion prior to compound treatment^15,16^. However, overnight incubation necessitates the use of medium supplemented with 10% FBS, which may reduce compound effectiveness (Supplementary Fig. 2) and inhibit luminescence signal, respectively. The presence of FBS completely prevented triciribine from enhancing FGF14:Nav1.6 complementation (10% FBS: 103.1 ± 8.9%, *n* = 8; 5% FBS: 97.6 ± 8.1%, *n* = 8; no FBS: 142.5% ± 10.7%, *n* = 8, *p* < 0.0001), and a higher concentration of FBS significantly reduced the potency of ZL181 (21.2 ± 2.8%, *n* = 8, p < 0.0001) compared to media with no FBS (11.2 ± 1.5%, *n* = 8, p < 0.0001). To circumvent this issue, as well as minimize potential variance associated with multiple liquid handling steps^35^, we attempted using cells in suspension by dispensing immediately prior to screening (cell-based homogeneous assay). Superior raw luminescence values (10446 ± 233.2 RLU, *n* = 8) were observed compared to adherent cells (8692 ± 78.7 RLU, *n* = 8, *p* < 0.0001, Supplementary Fig. S2). For these reasons, we find the use of cells in suspension to be superior to adherent cells for the purpose of increasing reliability and reducing costs. Additionally, we examined the effect of DMSO at varying concentrations (0.2–0.5% DMSO) on all cell densities and observed minimal effects at higher cell densities (Supplementary Fig. S2). Importantly, lower volumes reduce resource consumption, as well as increase well-capacity for subsequent assays (i.e., capacity for CTB assay reagent following LCA luminescence reading). We attempted to reduce the final 384-well volume by dispensing 20 µL of a 2X (6 mg/mL) luciferin solution, however higher luminescence and signal-background (S:B) separation was observed for those wells with 40 µL of 1X (3 mg/mL) luciferin (Supplementary Fig. S3).

**Figure 3:**
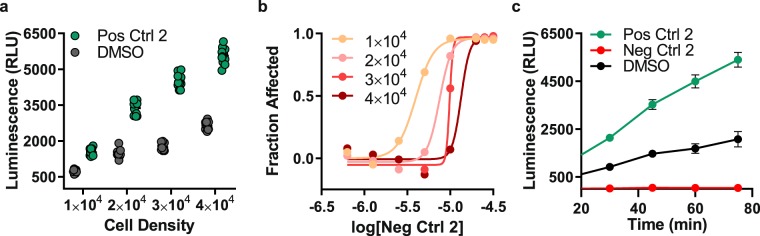
Cell density optimization in 384-well plates. (**a**) Luminescence values from Clone V cells treated with 0.3% DMSO (vehicle) or positive control 2 (50 ng/mL TNF-α) in 384-well plates containing cell densities ranging from 1–4 × 10^4^ cells/well. (**b**) Dose-response curves for negative control 2 (MNS; 8 concentrations: 0.625, 1.25, 2.5, 5, 10, 20, 25, and 30 µM, *n* = 6 per concentration) versus fraction affected, which corresponds to the proportion of Clone V cells that are inhibited by MNS treatment, in 384-well plates containing cell densities ranging from 1–4 × 10^4^. The MNS dose-response curve shifts to the left with decreasing cell density, indicating increased drug potency (MNS IC_50_: 1 × 10^4^, 3.96 µM; 2 × 10^4^, 7.49 µM; 3 × 10^4^, 9.82 µM; 4 × 10^4^, 13.3 µM). (**c**) Luminescence values from Clone V cells in 384-well plates (3 × 10^4^ per well**)** treated with either positive control 2 or negative control 2. Plate luminescence read in 15-minute intervals beginning 30 minutes after dispensing of luciferin substrate. Z′ was greatest for the 60-minute reading due to greater S:B ratio compared to earlier timepoints and lower SD compared to 75-minutes. Data shown are mean ± SD (*n* = 12 per treatment group).

Next, we optimized cell density per 384-well with respect to S:B ratio when treated with TNF-α. Cells were seeded at densities ranging from 1 × 10^4^–4 × 10^4^ cells per well, and luminescence was read following luciferin dispensing for up to 75-minutes, after which point the signal plateaus. We observed a positive linear relationship between luminescence and cell density for treatment with either 0.3% DMSO or 50 ng/mL TNF-α (Fig. 3a). The signal-background (S:B) ratio was significantly greater for a density of 3 × 10^4^ cells per well compared to densities of 1 × 10^4^, 2 × 10^4^, and 4 × 10^4^ cells per well (mean and SD: 2.60 ± 0.14 followed by 2.05 ± 0.11, 2.26 ± 0.18, and 2.12 ± 0.16X background signal, respectively; *n* = 16; p < 0.0001 using one-way ANOVA with post-hoc Tukey’s multiple comparisons). Additionally, we investigated the relationship between cell density and different doses (0.625, 1.25, 2.5, 5, 10, 20, 25, and 30 µM, *n* = 8 per concentration) of the negative control MNS (Fig. 3b). As expected, the dose-response curve shifts to the left with decreasing cell density, indicating increased drug potency (MNS IC_50_: 1 × 10^4^, 3.96 µM; 2 × 10^4^, 7.49 µM; 3 × 10^4^, 9.82 µM; 4 × 10^4^, 13.3 µM). For compound screening, each 384-well plate contains an 8-point dose-response of the negative control, enabling evaluation of plate-to-plate variability and rapid identification of faulty experiments. For instance, errors in cell plating leading to excess cells per well can be recognized by reduced potency of the negative control (dose-response curve shifted to the right).

Finally, we examined the effect of luciferin incubation time (Fig. 3c, Supplementary Fig. S3) on Z′ for cell densities ranging from 1 × 10^4^–4 × 10^4^ cells per 384-well. We observed that Z′ improves with increasing cell density (Table 1) and longer luciferin incubation (60 min; Fig. 3c) compared to earlier time points due to greater signal separation between positive and negative controls. However, Z′ stabilizes or decreases at later timepoints (75 min) due to increased SD of DMSO and TNF-α. Both 3 × 10^4^ and 4 × 10^4^ cells/well were sufficient to achieve a Z′ of 0.7 (Table 1); however, during compound screening, lower cell density translates into increased probability of a potent inhibitor to cross the hit threshold due to increased efficacy (Fig. 3b). Additionally, a 25% reduction in cell density substantially diminishes cell culture resource requirements when large volumes are required for HTS. Thus, the final optimized conditions for the assay in 384-well plates were as follows: cell density, 3 × 10^4^ per well; luciferin incubation time: 60 min; background control: 0.3% DMSO alone; positive control: 50 ng/mL TNF-α; and negative control: 25 µM MNS (Fig. 3c, Table 1).

**Table 1:** Z′-factor calculated for varying cell density and luminescence read timepoint.

Cell Density	Z′-factor					
	MNS-DMSO		TNF-DMSO		TNF-MNS	
	45 min	60 min	45 min	60 min	45 min	60 min
1 × 10^4^	0.41	0.43	−0.23	0.21	0.59	0.70
2 × 10^4^	0.63	0.51	0.36	0.37	0.75	0.75
3 × 10^4^	0.68	0.72	0.54	0.54	0.80	0.80
4 × 10^4^	0.58	0.62	0.54	0.54	0.82	0.82

Z′ values calculated (Eq. 1) for different cell densities (1–4 × 10^4^ cells per 384-well) at either 45- or 60-minutes following dispensing of luciferin substrate. For column 1 (MNS-DMSO), MNS was used as the positive control and DMSO as the negative control. For column 2 (TNF-DMSO), TNF-α was used as the positive control and DMSO as the negative control. For column 3 (TNF-MNS), TNF-α was used as the positive control and MNS as the negative control. Overall, the LCA is most robust across all categories using a cell density of 3 × 10^4^.

### Identification of novel regulators of the FGF14:Nav1.6 complex

We next tested this optimized system by screening a library comprised of 267 experimental or FDA-approved drugs from the CC_NCI collection. The compounds contained in this library have established toxicity profiles, are tolerable in humans, have well-established mechanisms of action and have been internally annotated with targets and cellular signaling pathways. Importantly, a subset of these compound’s targets overlap with pathways that our lab has previously explored using the transiently transfected FGF14:Nav1.6 system^16,19^, enabling us to directly compare and reconfirm previous results with this new assay.

**Figure 4:**
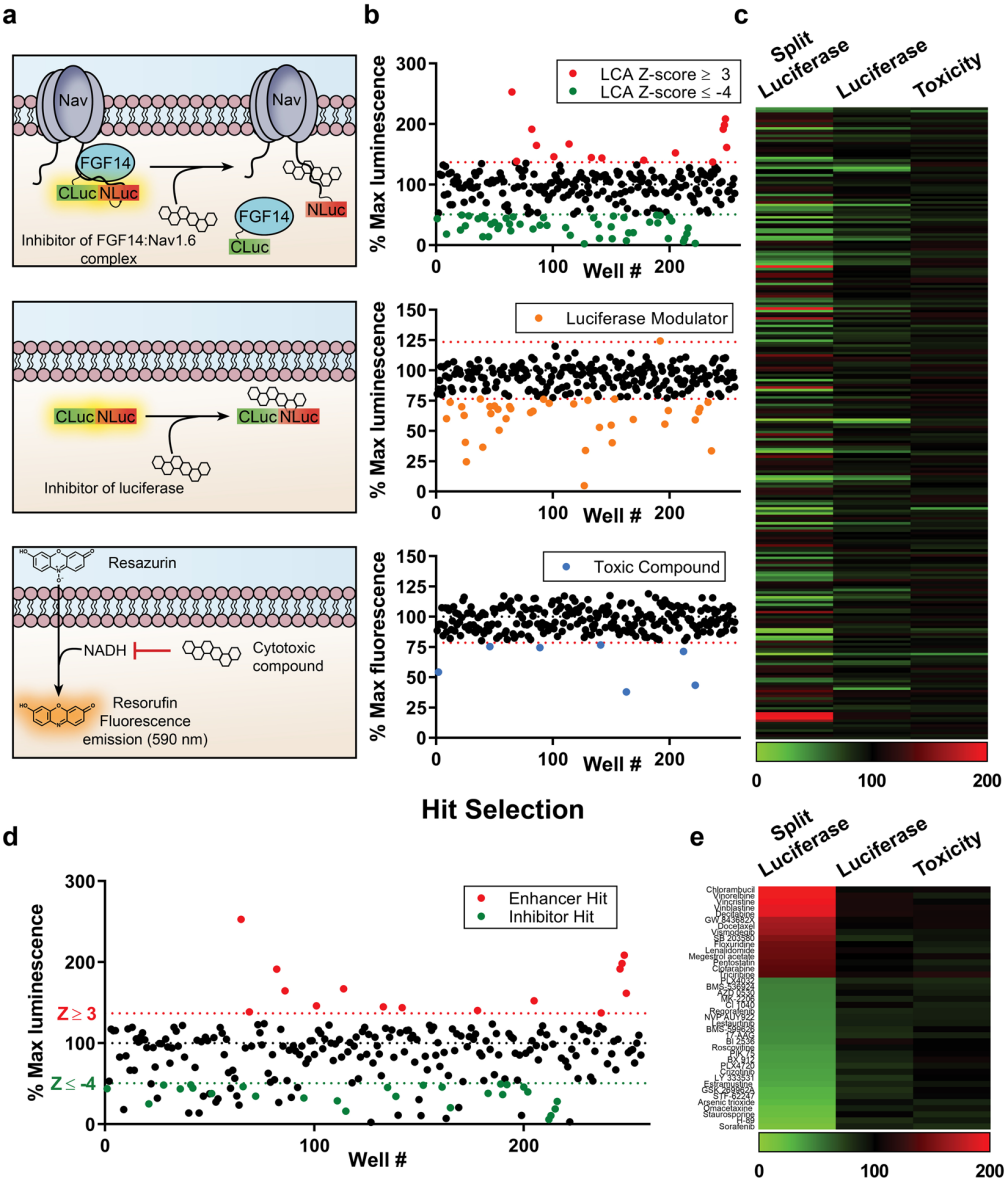
Identification of hits from the CC_NCI test library. (**a**) Cartoon representations of the primary assay and counter-screening assays used to identify false positives. Top: example of a compound that binds the Nav1.6 C-tail, preventing FGF14 binding and resulting in reduced complementation of luciferase fragments. Alternative mechanisms (not shown) include direct FGF14 binding or modulation of signaling pathways that regulate FGF14 or Nav1.6 through phosphorylation. Middle: example of a compound that reduces luminescence through direct inhibition of the luciferase enzyme, which could lead to false positives in the LCA. Bottom: example of a cytotoxic compound, leading to decreased NADH production and resulting in reduced fluorescence in the CTB cell viability assay. (**b**) Scatter plot of all compounds tested from the CC_NCI library showing % maximal luminescence or fluorescence (normalized to DMSO). Top: LCA results with preliminary hits highlighted as green (50 inhibitors; Z ≤ −4 and % max luminescence ≤ 50.7%) or red (15 enhancers; Z ≥ 3 and % max luminescence ≥ 137.0%). Middle: full-length luciferase assay in-cells used to identify false positives (Z ≥ ±3, equivalent to % max luminescence cut-offs of 76.6% and 123.4%, respectively); 1 enhancer and 36 inhibitors were identified, 22 of which were in the initial set of hits. Bottom: cell viability assay (CellTiter-Blue) identified 7 toxic compounds, 5 of which were in the initial set of hits (Z ≥ ±3, equivalent to % fluorescence cut-offs of 78.54% or 121.46%). Note: 2 of these toxic compounds were also inhibitors of the full-length luciferase. (**c**) Heat map representation of LCA, luciferase, and cell viability assay results. (**d,e**) Final hits were selected following exclusion of false positives and toxic compounds, resulting in a final set of hits including 14 enhancers and 26 inhibitors. (**d**) Scatter plot from (**a**) with final hits highlighted as green (inhibitors) or red (enhancers). (**e**) Heat map representation of final hits.

**Figure 5:**
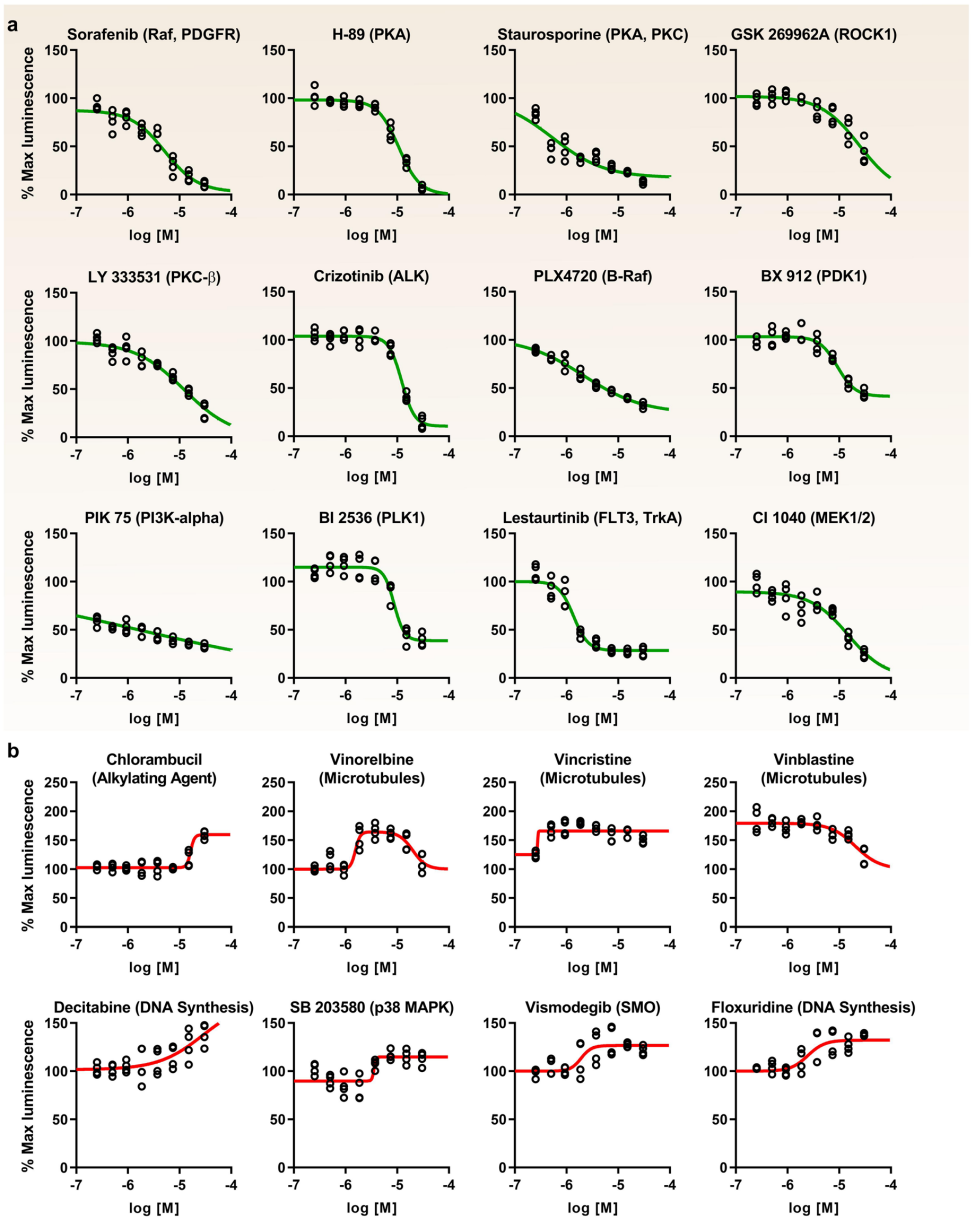
Initial dose-response validation of identified HTS hits. Graphs showing percent maximal luminescence from treated Clone V cells (normalized to DMSO controls) versus compound concentration with nonlinear regression curve fitting using Graphpad Prism 8. (**a**) Antagonists and (**b**) agonists are listed in order of efficacy as determined in the primary screening. Antagonists were defined as those compounds that inhibit FGF14:Nav1.6 complementation with increasing dose, while agonists were defined as those compounds which increase FGF14:Nav1.6 complementation with increasing dose. Estimated IC_50_ and EC_50_ values are provided in Table 2. Doses range from 0.25 µM–30 µM and were tested under identical conditions as the primary screening (*n* = 4 per concentration divided over two 384-well plates).

An overview of the protocol used for our screening is shown in Fig. 1c. Clone V cells were seeded in plates containing 0.3% DMSO (*n* = 16), cells alone (*n* = 8), 30 µM MNS (*n* = 8), MNS dose response (1.25, 2.5, 5, 7.5, 10, 15, 20, and 25 µM, *n* = 2 per concentration), and 50 ng/mL TNF-α (*n* = 16) controls and experimental compounds (30 µM; 1 compound per well). Z-scores (Eq. 5) were calculated for each compound using the mean and standard deviation of on-plate negative controls (0.3% DMSO). Immediately following luminescence reading, the cell viability assay was initiated by dispensing 10 µL of CTB reagent per well. Fluorescence was then read after 16 hrs, and cut-offs were set at a Z-score of <−3 to identify and exclude toxic compounds. This library was screened in duplicate, and the results are presented in Fig. 4. Initially, a total of 50 inhibitors and 15 enhancers were detected using Z-score cutoffs of +3 for enhancers and −4 for inhibitors, respectively. The cutoff for inhibitors was set such that no more than 50 candidate inhibitors were selected, which corresponds to Z-score < −4 and % maximal luminescence of 50.7%. Due to challenges in finding enhancers of Nav channels, a less stringent cutoff of Z-score > 3 was selected (corresponding to % max luminescence of 137%), resulting in 15 enhancers. Of these preliminary hits, 5 were excluded due to effects on cell viability (Z-score ≤ −3, equivalent to % fluorescence ≤ 78.54% of DMSO controls). Additionally, the library was counter-screened against the full-length luciferase (to identify potential false-positives) using transiently transfected HEK293 cells in 384-well plates under identical conditions as the primary assay. One luciferase enhancer and 36 luciferase inhibitors were identified in total, 22 of which were in the preliminary set of hits and excluded from further analysis. Interestingly, only one compound, PP121, significantly enhanced luminescence in the full-length luciferase assay (Z-score = 3.11), while inhibiting luminescence in the LCA (Z-score = −4.14). The effects of all compounds on the primary LCA, as well as the full-length luciferase and cell toxicity counter-screening assays are presented in Fig. 4 as percent luminescence (LCA and full-length luciferase assay) or fluorescence (cell viability assay) normalized to per plate DMSO controls (*n* = 16 per plate). To provide an integrated snapshot of the screening campaign, we represent the normalized response values in a heat-map (Fig. 4c,e, numerical data in Supplementary Table S1). Following exclusion of false positives identified in the counter screens (Fig. 4d), the set of hits included 15 enhancers and 25 inhibitors (Fig. 4e). From this initial set, 20 hits (12 inhibitors, 8 enhancers) were subsequently selected for follow-up based on LCA ranking and relevance of the drug target (Table 2). Hits were confirmed through an 8-point dose response (0.25, 0.5, 0.95, 1.88, 3.75, 7.5, 15, and 30 µM, *n* = 2 per concentration) in duplicate (20 hits per 384-well plate, *n* = 4 per concentration over two plates) (Fig. 5). Average normalized luminescence for each concentration and nonlinear curve fitting are shown in Fig. 5, and estimated IC/EC_50_ concentrations are provided in Table 2.

**Table 2:** Target-based hit assessment.

Rank	Antagonist	Target(s)	I_Min_ (% Luminescence)	Z-score	IC_50_ (µM)
1	Sorafenib	RAF, PDGFR, VEGFR2/3	5.98	−7.62	3.98
2	H-89	PKA	16.07	−6.81	12.16
3	Staurosporine	PKC, PKA, PKG	18.86	−6.58	0.54
4	GSK 269962 A	ROCK1	28.81	−5.77	24.66
5	LY 333531	PKCβ	34.34	−5.33	12.19
6	Crizotinib	ALK	34.72	−5.29	12.19
7	PLX4720	B-RafV600E	36.71	−5.13	2.14
8	BX 912	PDK1	37.84	−5.04	9.23
9	PIK 75	PI3Kα	38.11	−5.01	1.63
10	BI 2536	PLK1, BRD4	43.68	−4.57	8.71
11	Lestaurtinib	FLT3, JAK2	45.45	−4.42	1.22
12	CI 1040	MEK1/2	46.49	−4.34	14.85
**Rank**	**Agonist**	**Target(s)**	**E**_**Max**_ **(% Luminescence)**	**Z-score**	**EC**_**50**_ **(µM)**
1	Chlorambucil	Alkylating agent	252.88	12.39	16.41
2	Vinorelbine	Microtubules	208.53	8.79	1.55
3	Vincristine	Microtubules	198.49	7.99	0.27
4	Vinblastine	Microtubules	191.58	7.42	20.56
5	Decitabine	DNA synthesis inhibitor	191.40	7.41	28.77
6	Vismodegib	SMO	161.54	4.99	1.91
7	SB 203580	p38 MAPK	152.42	4.25	3.56
8	Floxuridine	Antimetabolite	146.01	3.73	2.46

Hits are ranked by average Z-score from the primary screening (*n* = 2). Estimated IC_50_ and EC_50_s are calculated from data represented in Fig. 5.

We found that the FGF14:Nav1.6 C-tail interaction was indirectly inhibited (i.e., the relevant kinase inhibitor acts as antagonist) through targeting S/T kinases including rapidly accelerated fibrosarcoma (c-RAF), protein kinases A, C, and G (PKA, PKC, PKG), rho-associated coiled-coil-containing protein kinase 1 (ROCK1), pyruvate dehydrogenase kinase 1 (PDK1), phosphoinositide 3-kinases (PI3K), polo-like kinase 1 (PLK1), and mitogen-activated protein kinase kinase (MEK1, aka MAP2K1). RAF kinases participate in the RAS-RAF-MEK-ERK signal transduction cascade^36^; this pathway likely stimulates the FGF14:Nav1.6 interaction, as inhibition of RAF, MEK1, and p38 MAPK (lower doses of SB 203580, Fig. 5) all reduced this interaction in our assay. While non-specificity (i.e., off-target effects) is a common issue for experiments involving kinase inhibitors, the observation of multiple inhibitors targeting numerous kinases in the same pathway lends support to these results. Additionally, inhibition of the following protein or receptor tyrosine kinases (RTKs) reduces PPI between FGF14 and Nav1.6 C-tail: platelet-derived growth factor receptor (PDGFR), vascular endothelial growth factor receptor (VEGFR) 2 and 3, anaplastic lymphoma kinase (ALK), FMS-like tyrosine kinase 3 (FLT3), Tropomyosin receptor kinase A (TrkA), and janus-associated kinase 2 (JAK2). Interestingly, numerous DNA synthesis inhibitors (anti-metabolites), alkylating agents, and microtubule inhibitors enhanced the FGF14:Nav1.6 interaction; while exploration of possible mechanisms for these compounds are subjects for future investigation, the results may not be biologically relevant for neuronal Nav channel function. The p38 MAPK inhibitor SB 203580 was initially found to enhance the FGF14:Nav1.6 interaction in the primary screening (30 µM), but evaluation of dose-dependent behavior (Fig. 5) revealed mild inhibition at lower concentrations (0.5–2 µM) and stimulation at higher concentrations, indicative of off-target effects. ROCK1 is a is a regulator of the actomyosin cytoskeleton which promotes contractile force generation^37^; this finding in combination with the numerous microtubule hits observed in our assay serves to reinforce the idea that the cytoskeleton may play a role in controlling FGF14:Nav1.6 interactions.

**Figure 6:**
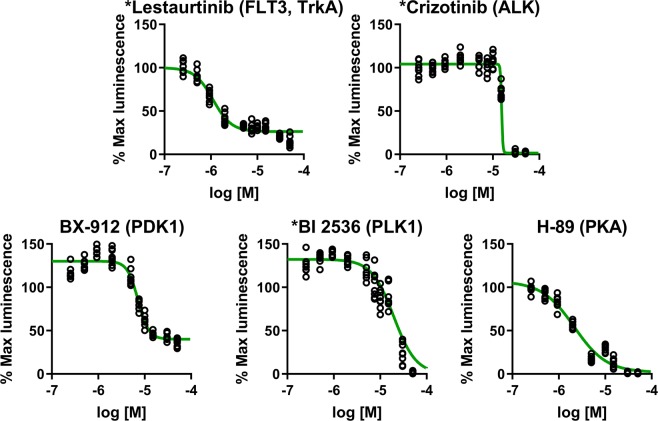
Second validation of prioritized hits using repurchased compounds. Based on initial dose responses (potency, efficacy, and curve shape) as well as target information, the tyrosine kinase inhibitors Lestaurtinib and Crizotinib, as well as the S/T kinase inhibitors H-89, BX-912, and BI 2536, were repurchased to confirm compound identity and potency. Fresh compounds were tested by 10-point dose responses (range: 0.25 µM–50 µM) in Clone V cells under identical conditions as the primary screening. All compounds demonstrated results similar to the original. While Lestaurtinib, Crizotinib, and H-89 appear to have purely inhibitory effects, BX-912 and BI 2536 act as enhancers at lower concentrations. Estimated IC_50_: Lestaurtinib, 0.95 µM; Crizotinib, 15.5 µM; H-89, 1.9 µM; BX-912, 6.8 µM; BI 2536, 17.4 µM. *Drug has completed and/or on-going clinical trials, including for PNS or CNS-related cancers.

Based on initial dose responses (potency, efficacy, and curve shape) as well as target information, 5 compounds (Lestaurtinib, Crizotinib, H-89, BX-912, and BI 2536) were repurchased to confirm compound identity and establish potency. The freshly acquired compounds were retested in Clone V cells using 10 doses (0.25, 0.5, 1, 2.5, 5, 7.5, 10, 15, 30, and 50 µM, *n* = 8 per concentration) in duplicate (10 compounds per 384-well plate, *n* = 8 per concentration over two plates). Results confirm the dose-dependent activity of all compounds, which are similar to the primary screen (Fig. 6). The most potent inhibitor identified by this screen was Lestaurtinib, with an in-cell IC_50_ of 0.95 µM, followed by H-89 (1.9 µM), BX-912 (6.8 µM), Crizotinib (15.5 µM), and BI 2536 (17.4 µM). While Lestaurtinib, Crizotinib, and H-89 appear to have purely inhibitory effects, BX-912 and BI 2536 display counter acting activity as a function of dosage. However, the sigmoidal appearance of dose-response curves is promising. Based on this data, we identify these five inhibitors as top hits from our screening against the FGF14:Nav1.6 complex and recommend follow-up functional studies to determine the effects of the relevant kinase targets on neuronal excitability. Overall, these results demonstrate that: (1) our assay is capable of reliably detecting both inhibitors and enhancers of the FGF14:Nav1.6 complex from a background signal with low variability; (2) initial effects of identified hits can be reproduced in subsequent studies; and (3) that the screening system is capable of follow-up dose-dependency studies using an identical 384-well plate format with additional replicates for each concentration.

## Discussion

Despite extensive interest in pharmacologically targeting protein-channel complexes^11,38–41^, the lack of adequate platforms to rapidly screen compounds in physiologically relevant models^42,43^ has significantly hampered discovery of compounds targeting these interfaces. Growing appreciation of how ion channels and receptors operate as macromolecular complexes, rather than isolated entities within the lipid bilayer, necessitates drug development strategies beyond conventional agonists and antagonists targeting voltage-sensitive domains or ligand binding pockets^44,45^. Mutations that impact the intracellular portions of the Na^+^, K^+^, and Ca^2+^ channel’s pore-forming alpha subunits or their accessory proteins are associated with genetically inherited epilepsies^46^. However, given that the effects of these mutations are heterogenous and have variable impact on patients, there is a dearth of uniform or efficacious treatments. Increasing evidence suggests that specific microdomains separate from the ion permeating region of these channels are associated with physiologically- and disease-relevant effects and could possibly be targeted as allosteric surfaces for drug development. It is therefore necessary to develop strategies to study these ancillary portions of the primary channel with precise and targeted methods.

One of the challenges in searching for ion channel regulators is that these protein complexes, particularly for Nav channels, are large and difficult to reliably express in heterologous cells using lipid-based transfection. Traditionally, whole cell patch-clamp has been used as a functional readout of channel activity; however, this technique does not easily transition to HTS when the channel is assessed in the presence of an accessory protein. This is in part due to the lack of ability to control protein-channel interactions during the channel cycle within multi-well plates^47^ and to specifically isolate these interactions from the rest of the channel.

Here, we have applied a minimal functional domain (MFD) approach to isolate specific regions within Nav channels^48^. Our strategy isolates the MFD within the FGF14:Nav1.6 channel complex, reconstitutes this domain in a heterologous cell system, and uses LCA to investigate specific interactions while maintaining the protein:channel domain near-to-physiological conditions. The design of the CD4-Nav1.6 C-tail chimera anchors the C-tail to the inner leaf of the plasma membrane, enabling closer to native presentation of the FGF14:Nav1.6 interacting domain compared to diffuse and freely floating cytosolic Nav1.6 C-tail. For screening modulators of cell signaling, maintaining these interacting proteins in membrane microdomains increases the likelihood of identifying the most physiologically relevant Nav1.6 regulatory pathways.

Building on previous studies in which LCA was conducted using transient transfection, here we created a double stable cell line that expresses the FGF14:Nav1.6 C-tail complex and miniaturized this assay from 96- to 384-well plates to be amenable for HTS of large chemical libraries. Our new assay platform implements liquid-handling robotic systems, enabling rigorous counter-screens to be conducted in parallel with LCA, which drastically reduces false positives while simultaneously allowing for expedient hit validation studies. Using 3 × 10^4^ cells per 384-well in suspension and 1 hr reporter substrate incubation, our assay achieved Z′ > 0.5 for both inhibitory and enhancer-type assays (Table 1, columns 1 and 2) and exhibited a robust ability (Z′ > 0.8) to distinguish agonist from antagonist (Table 1, column 3). Thus, this miniaturized LCA is capable of reliably distinguishing significant FGF14:Nav1.6 modulators from background signal.

Using this new miniaturized assay, we screened a test library of 267 FDA-approved and clinical oncology drugs and identified potent agonists and antagonists of the FGF14:Nav1.6 interaction (Table 2). The rationale for selecting this library was two-fold. First, the compounds target a broad range of cell signaling pathways potentially important for regulation of the Nav channel complex while simultaneously screening clinically relevant compounds that could be repurposed for CNS disorders and channelopathies. Second, the economical size of the library (one 384-well plate including controls) facilitated duplicate screening under numerous conditions throughout development, enabling extensive assay optimization prior to larger campaigns. The initial hit selection criteria were based on previously identified challenges in detecting potent enhancers of the FGF14:Nav1.6 C-tail interaction^19,49^, and the target profile and chemical attributes of compounds were subsequently analyzed to determine top hits for validation studies (Fig. 1c and Table 2). These data show that many of the hits target kinases that are known to play an important role in regulating PPI that affect electrical activity of neurons^5^. The PI3K/Akt pathway, which converges on GSK3, has been identified as a prospective regulatory node of neuronal excitability through modulation of the FGF14:Nav1.6 complex^19^. GSK3β directly phosphorylates FGF14 at S226 and Nav1.6 at T1936, two sites that were found to be disease-related in experimental models of neurodegeneration and of vulnerability to stress and depression, respectively^27,32^. Clusters of S/T kinase inhibitors, including those targeting CK2, PKC and Wee1 kinase, have been found to converge on the FGF14:Nav1.6 complex through the GSK3 pathway^19^. For example, inhibitors of CK2, which serves as a priming kinase for GSK3 in neurons and has been shown to phosphorylate FGF14 at S228 and S230, are strong suppressors of the FGF14:Nav1.6 interaction and decrease excitability in hippocampal neurons^27^. Thus, it is possible that hits identified in this study would modulate the FGF14:Nav1.6 complex through finely-tuned regulation of phosphorylation at these sites. Based on this information, we selected five ‘hit’ kinase inhibitors for extensive dose-dependency validation studies using fresh compound samples, including H-90, Critzotinib, BX913, Lestaurtinib, and BI2537, which all acted as antagonists toward the FGF14:Nav1.6 C-tail interaction. These kinases also converge on the Akt/GSK3 pathway, which alters Nav1.6 current^19^ and modulates neuronal excitability and leads to various behavioral outcomes^32^. For example, activation of PKA reduces Nav1.6 currents in heterologous cell systems^50^, and disruption of the PDK1–Akt pathway leads to cognitive deficits and diminished motivation^51^. Additionally, ALK-PI3K pathway plays a role in learning, memory and neurogenesis^52^ and synaptic plasticity in the nucleus accumbens^53^.

Importantly, we show that the FDA-approved drug lestaurtinib might be of interest for regulating excitability in channelopathies. This tyrosine kinase inhibitor targets the JAK2^54^, FLT3, and TrkA pathways and is the most potent inhibitor (IC_50_ = 0.95 μM) of the FGF14:Nav1.6 interaction that we have identified to date. There are currently 14 ongoing or completed clinical trials using lestaurtinib for the treatment of various cancers including myelofibrosis, leukemia, prostate cancer, and neuroblastoma, and the TrkA pathway is a target for neuroblastoma therapy^55^. Furthermore, the brain-derived neurotrophic factor (BDNF)-TrkB pathway has been implicated in channelopathies^56^, and inhibition of this pathway by lestaurtinib prevents epileptogenesis in immature brains^57^ and hyperexcitability-induced emotional and cognitive behavioral dysfunction after hypoxic seizures^58^. These results indicate that this FDA-approved drug might be of interest for CNS activity in diseases characterized by dysfunction of Trk receptor signaling. Our results suggest that lestaurtinib could be potentially repurposed toward channelopathies and other CNS diseases characterized by dysfunction of neuronal excitability mediated by Nav1.6. However, these results await functional validation studies, such as electrophysiology, and extensive *in vivo* studies for further validation.

In summary, here we report a robust assay for HTS of small molecules based on split-luciferase complementation that could be applied to search for mechanisms regulating ion channel complexes and develop targeted treatments for channelopathies associated with changes in protein:channel interactions. In addition to repurposing FDA-approved anti-cancer therapeutics as described here, this platform is amenable for targeted campaigns using small molecule libraries against hot-spots at the protein:channel interface. These efforts could lead to allosteric modulators of Nav channels through a traditional lead optimization phase, including a cascade of orthogonal screenings, functional assays (i.e., electrophysiology) and behavioral pharmacology^45^. Overall, we anticipate that our MFD driven platform^59^ could provide the foundation for development of new classes of protein:protein interaction-based leads to treat channelopathies and other CNS disorders.

## Methods

### DNA constructs

Mammalian expression vectors coding for N-terminal (pcDNA3.1-V5_HIS TOPO; rapamycin-binding domain [FRB]-N-terminal luciferase fragment [FRB-NLuc]) and C-terminal (pEF6-V5_HIS TOPO; C-terminal luciferase fragment [CLuc-FKBP]) fragments of firefly (*Photinus pyralis*) luciferase were a gift of Dr. Piwnica-Worms (University of Texas MD Anderson, Houston, TX) from pioneering studies^60^. To generate the CLuc-FGF14 construct, FKBP was replaced with neuronal FGF14 (1b isoform) in the CLuc-FKBP fusion vector. CLuc-FGF14 was engineered by polymerase chain reaction (PCR) amplification of the FGF14 open reading frame (nt 1–855) using a 50-nt primer containing a BsiWI site up to a linker region and a 30-nt primer containing a NotI site and ligated into the CLuc vector. To generate the CD4-Nav1.6-NLuc construct, a chimera carrying the C-terminal fragment of Nav1.6 (amino acids 1763–1976) fused with CD4ΔC-tail (amino acids 1–395; gift of Dr. Benedict Dargent, INSERM, France) similarly replaced FRB in the FRB-NLuc construct using PCR amplification and ligation into BamHI at the 50 end and BsiWI at the 30 end. The choice of using the CD4 chimera fused to Nav1.6 C-tail was based on previous validations of this and other similar constructs in primary hippocampal neurons^61–63^. Because the N-terminus of the Nav channels is located intracellularly, the fusion of the NLuc fragment to the Nav1.6 C-tail resulted in intracellular reconstitution of the two halves of luciferase. The CLuc-FGF14 DNA was sub-cloned in pcDNA4-TO-Puromycin vector (Addgene) at BamHI/NotI sites. The CLuc-FGF14 fragment was amplified using specific primers having restriction sites in forward BamHI and NotI in reverse primer. The putative clones were screened by colony PCR, restriction digestion. To remove ECFP from pcDNA4-TO-Puro-CLuc-FGF14-ECFP, we added XbaI site at N-terminal of ECFP with site-directed mutagenesis. The pcDNA4-TO-Puro-CLuc-FGF14-ECFP was digested with XbaI to remove ECFP fragment and linear pcDNA4-TO-Puro-CLuc-FGF14 construct was further ligated using T4-DNA ligase (NEB). Finally, the construct pcDNA4-TO-Puro-CLuc-FGF14 was verified by sequencing at UTMB core facility.

### Cell culture

HEK293 cells were incubated at 37 C with 5% CO_2_ in medium composed of equal volumes of Dulbecco modified essential medium (DMEM) and F12 (Invitrogen, Carlsbad, CA) supplemented with 10% fetal bovine serum, 100 U/mL penicillin, and 100 mg/mL streptomycin. For transfection, cells were seeded in 24-well CELLSTAR^®^ tissue culture plates (Greiner Bio-One, Monroe, NC) at 4.5 × 10^5^ cells per well and incubated overnight to give monolayers at 90%–100% confluency. Cells were then transiently transfected with the CLuc-FGF14 and CD4-Nav1.6-C-tail-NLuc constructs or the *Photinus pyralis* luciferase construct (pGL3) using Lipofectamine 2000 (Invitrogen), as described previously^15,19,49,64^.

To generate the HEK293 double stable cell line expressing both Nav1.6 C-tail and FGF14, linearized pcDNA3-CD4-Nav1.6C-tail-NLuc constructs (Fig. 1b) were transfected into HEK293 cells as described previously^15,19,49,64^. Cells were grown with 0.5 mg/mL geneticin G418 (also known as Neomycin; Life Technologies) for 1–2 weeks, and single healthy clones were selected and expanded for 3–4 week cycle. The single stable line was validated by transiently transfection of CLuc-FGF14 and subsequent luminescence reading in 96-well plates (see below). Next, we transfected linearized pcDNA4-TO-Puro-CLucFGF14 plasmid into a single stable clone of pcDNA3-CD4-Nav1.6 C-tail-NLuc HEK293 cells. Single healthy clones were selected and expanded for another 3–4-week cycle under 2.5 µg/mL puromycin. The newly generated double stable clone was maintained under 0.5 mg/mL G418 and 2.5 µg/mL puromycin.

### Split-luciferase complementation assay

#### 96-well plate assay

Cells were trypsinized (0.25%), triturated in a medium, and seeded in white, clear-bottom CELLSTAR μClear^®^ 96-well tissue culture plates (Greiner Bio-One) at ∼10^5^ cells per well in 200 μL of medium.

For transiently transfected cells, trypsinization was performed 48 h post-transfection. The cells were incubated for 24 h, and the growth medium was subsequently replaced with 100 μL of serum-free, phenol red–free DMEM/F12 medium (Invitrogen) containing protein kinase inhibitors (1–50 μM) or TNF-α (1–50 ng/mL). The final concentration of DMSO was maintained at 0.3% or 0.5% for all wells excluding the positive control wells containing medium alone. Following 2 h incubation at 37 C, the reporter reaction was initiated by injection of 100 μL substrate solution containing 1.5 mg/mL of D-luciferin dissolved in PBS (final concentration = 0.75 mg/mL) by the Synergy™ H4 Multi-Mode Microplate Reader (BioTek, Winooski, VT). Luminescence readings were performed at 2-min intervals for 20–30 min, integration time 0.5 s, and the cells were maintained at 37 °C throughout the measurements. Signal intensity for each well was calculated as a mean value of peak luminescence; the calculated values were expressed as percentage of mean signal intensity in the control samples from the same experimental plate. For our assay, an enhancer is defined as a positive modulator and inhibitor is defined as a negative modulator of FGF14:Nav1.6 complex assembly, as detected through increases or decreases in luminescence, respectively.

#### 384-well plate assay

Cells were trypsinized (0.25%), triturated in a medium, and seeded in white, clear-bottom CELLSTAR μClear® 384-well tissue culture plates (Greiner Bio-One) at 30 000 (3 × 10^4^) cells per well in 40 μL of serum-free, phenol red–free DMEM/F12 medium using the Multidrop Combi (Thermo Fisher). Nanoliter volumes of library compounds or controls (DMSO, TNF-α, MNS) are transferred from low dead volume source plates using the LabCyte Echo 550 acoustic transfer platform. The final concentration of DMSO was maintained at 0.3% for all wells excluding those wells containing cells with medium alone. Following 2 h incubation at 37 C, the reporter reaction was initiated by injection of 40 μL substrate solution containing 1.5 mg/mL of D-luciferin (final concentration = 0.75 mg/mL) by the Multidrop Combi. After 1 h incubation, luminescence intensity is read using a Tecan Infinite M1000.

D-luciferin was purchased from BioGold and prepared as a 30 mg/mL stock solution in phosphate- buffered saline (PBS). Recombinant human TNF-α was purchased from Abcam and dissolved in PBS containing 0.1 mg/mL BSA (for protein stability) and prepared as a 10 µg/mL stock solution. Triciribine and MNS were purchased from Tocris, and ZL181 was developed by the laboratory of Jia Zhou at UTMB; each drug was dissolved in dimethyl sulfoxide (DMSO) as 20 mM stock solutions. Drugs for the HTS were provided as 10 mM stock solutions in DMSO on 384-well plates.

### Cell viability assay

As a counter screen, we performed a cell viability analysis using CellTiter-Blue (CTB, Promega). Immediately following luminescence reading, 10 µL of 1X CTB reagent was dispensed into 384-well plates using the Multidrop Combi; plates were incubated overnight (16 h) at 37 C, and fluorescence was detected using the Tecan Infinite M1000 reader (excitation λ = 560 nm, emission λ = 590 nm). Cell viability was expressed as percent of the mean fluorescent signal intensity of on-plate negative controls.

### Western blotting

Cell lysates were collected after treatment of Clone V cells with 25 µM MNS and 50 ng/µL TNF-α for 2 hrs, resolved on 12% SDS-PAGE, transferred to PVDF membranes, and visualized as previously described^49,63^. The following antibodies were used: anti-luciferase (epitope detects 251–550 aa) (Santa Cruz Biotechnology), anti-α-tubulin (#12G10, U.Iowa DSHB)^49,63,64^, and anti-TNFR1 H-5 (Santa Cruz Biotechnology).

### Data analysis

Statistical parameters of assay performance were calculated according to the following formulas:

1 $${\rm{Z}}^{\prime} \,{\rm{factor}}=1-3\times \frac{({\delta }_{{\rm{p}}}+{\delta }_{{\rm{n}}})}{({\mu }_{{\rm{p}}}-{\mu }_{{\rm{n}}})}$$

2 $${\rm{S}}:\,{\rm{B}}=\frac{{\mu }_{{\rm{p}}}}{{\mu }_{{\rm{n}}}}$$

3 $${\rm{S}}:\,{\rm{N}}=\frac{({\mu }_{{\rm{p}}}-{\mu }_{{\rm{n}}})}{\sqrt{{\sigma }_{{\rm{p}}}^{2}+{\sigma }_{{\rm{n}}}^{2}}}$$

4 $${\rm{SW}}=\frac{{\mu }_{{\rm{p}}}-{\mu }_{{\rm{n}}}-3\times ({\sigma }_{{\rm{p}}}+{\sigma }_{{\rm{n}}})}{{\sigma }_{{\rm{p}}}}$$

where δ_p_ and δ_n_ are standard deviation of the positive control group p and the negative control group n, and μ_p_ and μ_n_ are the arithmetic means of the two groups, respectively; S:B, signal to background; S:N, signal-to-noise; and SW, signal window^47^. For cell-based assays, a Z′ of ≥0.5 signifies that outliers can be reliably identified as statistically significant despite well-to-well and plate-to-plate variability. We calculated Z′ using Eq. 1, which is based on the mean and standard deviation of positive and negative controls and is calculated in the absence of library compounds^65^. Based on this equation, Z′ is improved by greater signal separation between the mean of positive and negative controls, as well as by reducing variance between replicates (i.e., standard deviation). In practical terms, consistency between replicates would improve confidence in a single well outlier being truly significant (i.e., the compound treatment in that single well resulted in significant changes in complex formation, rather than the change in luminescence being due to simple well-to-well variability).

5 $${\rm{Z}}\,{\rm{score}}=\frac{{\mu }_{i}-{\mu }_{DMSO}}{{\mu }_{DMSO}}$$

where μ_i_ and μ_DMSO_ are the arithmetic means of the sample (i.e., screened compound) and 0.3% DMSO control group, respectively.

Dose-response curves were obtained using GraphPad Prism 8 by fitting the data with a non-linear regression:

6 $$A+\frac{B-A}{1+{10}^{\log ({x}_{0}-x)}H}$$

where x is log_10_ of the compound concentration in M, x_0_ is the inflection point (EC_50_ or IC_50_), A is the bottom plateau effect, B is the top plateau effect, and H is the Hill slope. Kinase inhibitors that increased FGF14:Nav1.6 interaction with increasing doses were classified as agonists; inhibitors that decreased FGF14:Nav1.6 interaction were classified as antagonists.

## Supplementary Information

Supplementary Information
