# Supplementary material for: High-throughput screening against protein:protein interaction interfaces reveals anti-cancer therapeutics as potent modulators of the voltage-gated Na+ channel complex
Source: Sci Rep. 2019 Nov 15;9:16890. doi: 10.1038/s41598-019-53110-8 (PMC6858373; doi:10.1038/s41598-019-53110-8)
Supplement: Supplementary file 1 — Supplementary Information [file 41598_2019_53110_MOESM1_ESM.docx]

**High-throughput screening against protein:protein interaction interfaces reveals anti-cancer therapeutics as potent modulators of the voltage-gated Na^+^ channel complex**

Paul A. Wadsworth^1,2,+^, Oluwarotimi Folorunso^2,+^, Nghi Nguyen^3^, Aditya K. Singh^2^, Daniela D’Amico^4^, Reid T. Powell^3^, David Brunell^3^, John Allen^2^, Clifford Stephan^3^, Fernanda Laezza^2,^*

^1^Biochemistry and Molecular Biology Graduate Program and ^2^Department of Pharmacology & Toxicology, The University of Texas Medical Branch, Galveston, Texas 77550, USA;

^3^HTS Screening Core, Center for Translational Cancer Research, Texas A&M Health Science Center: Institute of Biosciences and Technology, Houston, TX 77030, USA;

^4^Neuroscience Graduate Program, The University of Texas Medical Branch, Galveston, Texas 77555, USA;

^+^these authors contributed equally to this work

*correspondence to felaezza@utmb.edu

**
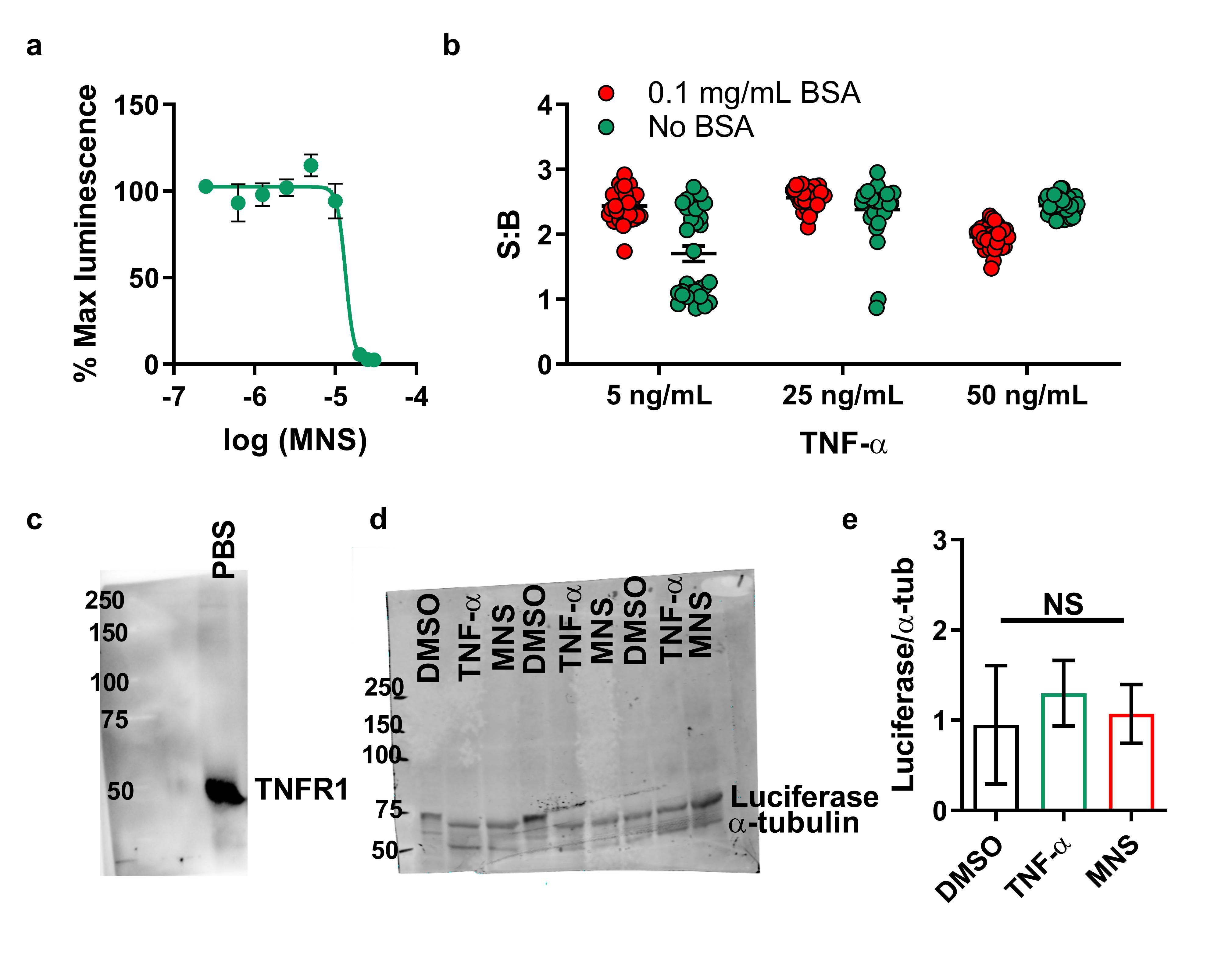
**

**Supplementary Figure S1. Validation of TNF-α and MNS as LCA controls and confirmation of TNFR1 expression in HEK293 cells.** (**A**) Plot of percent luminescence (normalized to DMSO controls) from Clone V cells treated with MNS in 384-well plates versus compound concentration (range: 0.625 - 25 μM, *n=*16 per concentration) with nonlinear regression curve fitting using Graphpad Prism 8. Estimated IC_50_ = 13.35 μM. Based on these results, we used a final concentration of 30 μM for the 384-well plate inhibitory controls that were used to calculate Z’. (**B**) Plot of signal to background (S:B) ratio from Clone V cells treated with recombinant human TNF-α (positive control 2) in 384-well plates (*n=*32 per treatment group), calculated using DMSO controls as the background signal. The manufacturer recommends to supplement TNF-α with 0.1 mg/mL BSA to improve stability; however, the increased viscosity of this solution was problematic for dispensing using the LabCyte Echo 550. We found that BSA decreased the variance between replicates at lower concentrations of TNF-α (5 and 25 ng/mL), but that using a higher concentration of TNF-α without BSA could replicate this result. This point highlights the necessity of carefully examining practical concerns such as solution viscosity for high-throughput screening. (**C**) Immunoblot with antibody against human TNFR1 on lysate of double stable HEK293 cells. Blot was cropped to remove bands not pertaining to this paper (**D,E**) Immunoblot with anti-Luciferase (251-550 aa) and α-tubulin (as control for protein loading) on lysate of double stable HEK293 cells treated with 50 ng/mL TNF-α (positive control 2) or 30 μM MNS for 2 hrs. (**E**) Quantification of bands in (**B**); data are mean ± SEM (*n=*3 per treatment group). One-way ANOVA with post-hoc Dunnett’s multiple comparisons test was used to determine significance.


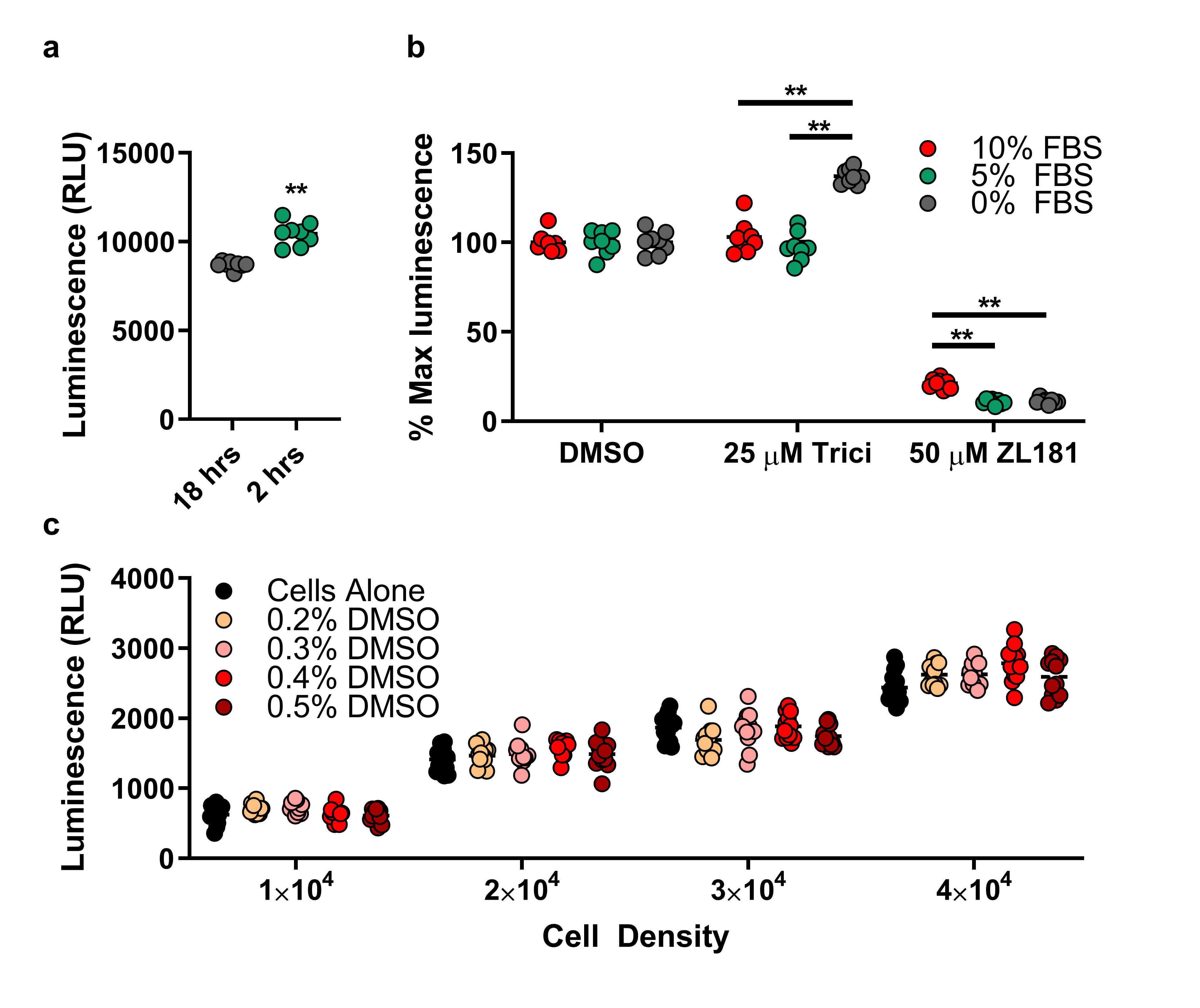


**Supplementary Figure S2. Effect of cell adhesion, FBS, and DMSO on assay performance.** (**A**) Raw luminescence from Clone V cells plated either 18 hrs (adherent) or 2 hrs (suspension) prior to luminescence reading. The luminescence was significantly higher for cells in suspension, indicating that successful luciferase complementation does not require complete cell adherence. Unpaired t test was used to determine significance; p<0.0001. (**B**) Stable cells in plated 2 hrs prior to plate reading in media containing either 10%, 5%, or no FBS were treated with either 0.3% DMSO (control), 25 uM triciribine, or 50 uM ZL181. The effect of triciribine was abolished in media containing FBS, while the effect of ZL181 was similar in 5% FBS but reduced in 10% FBS. *n=*8 per treatment group. Two-way ANOVA with post-hoc Tukey’s multiple comparisons test was used to determine significance; p<0.0001. (**C**) Raw luminescence from Clone V cells in 384-well plates in media containing 0.2%, 0.3%, 0.4%, or 0.5% DMSO or without DMSO. *n=*12 per treatment group

**Supplementary Figure S3. Effect of luciferin incubation time and dispensed luciferin volume on luminescence signal.** Raw luminescence values from Clone V cells plated in 384-well plates at different cell densities (1-4×10^4^) treated with 0.3% DMSO alone (control) or 50 ng/mL TNF-α (positive control 2). Graphs show luminescence reading after (A) 30 mins, (B) 45 mins, and (C) 60 mins of luciferin incubation. The luminescence signal increases over time and with increasing cell density, and the signal plateaus after 1 hr (not shown). **
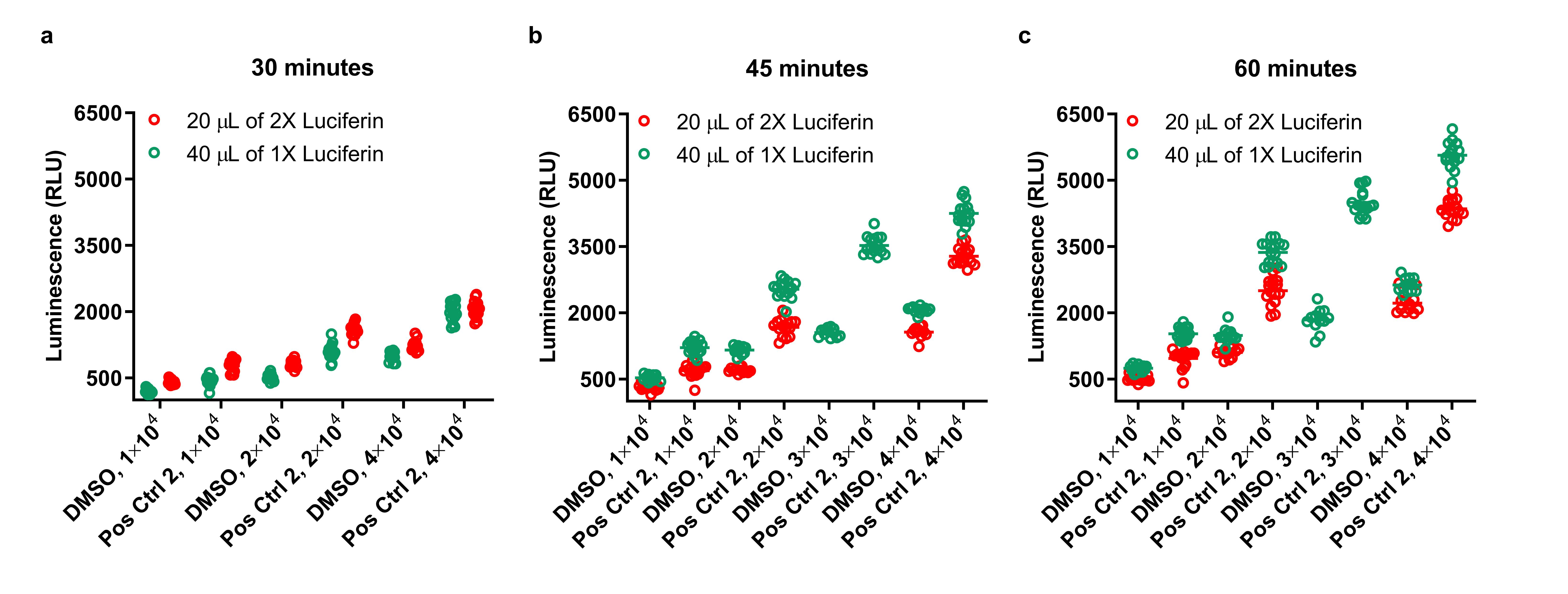
**Although the resulting final concentration of luciferin per 384-well (1.5 mg/mL) was in theory identical after dispensing either 20 µL of 2X (6 mg/mL) or 40 µL of 1X (3 mg/mL) luciferin dissolved in PBS, the higher volume enabled production of greater luminescence and was used as the final condition for our assay. Lower volumes may be desirable to reduce resource consumption, as well as to increase well-capacity for subsequent assays (i.e., dispensing 30 µL of CTB reagent to initiate the cell viability assay immediately after LCA luminescence reading).

**Supplementary Table S1.** All compound data from the CC_NCI screening, including the LCA luminescence reading (% Lum) over two biological replicates (Set 1 and Set 2), the CTB cell viability assay fluorescence reading (% Fluor), and the full-length luciferase counter-screening luminescence reading (% Lum). All data, including Z-scores, are normalized to corresponding on-plate controls (0.3% DMSO, *n*=16 per plate).

|  | **Split Luciferase Assay** | | | | | **Toxicity Assay** | | **Luciferase Assay** | |
| --- | --- | --- | --- | --- | --- | --- | --- | --- | --- |
| **Compound** | **% Lum Set 1** | **Z-score**  **Set 1** | **%Lum Set 2** | **Z-score Set 2** | **%Lum Avg** | **% Fluor** | **Z-score** | **% Lum** | **Z-score** |
| ABT-263 | 93.82 | -0.50 | 71.78 | -2.30 | 82.80 | 92.92 | -0.99 | 103.24 | 0.42 |
| AXITINIB | 107.95 | 0.64 | 91.55 | -0.69 | 99.75 | 91.58 | -1.18 | 40.56 | -7.61 |
| ABT-869 | 14.46 | -6.91 | 21.85 | -6.36 | 18.15 | 87.41 | -1.76 | 60.04 | -5.12 |
| 17-AAG | 45.98 | -4.37 | 41.61 | -4.75 | 43.80 | 88.49 | -1.61 | 80.45 | -2.50 |
| AZD 0530 | 36.20 | -5.16 | 60.34 | -3.23 | 48.27 | 89.05 | -1.53 | 98.71 | -0.17 |
| BORTEZOMIB | 55.77 | -3.57 | 84.27 | -1.28 | 70.02 | 89.20 | -1.51 | 88.79 | -1.44 |
| BIBF-1120 | 119.91 | 1.61 | 123.80 | 1.94 | 121.85 | 89.46 | -1.47 | 102.06 | 0.26 |
| BOSUTINIB | 109.04 | 0.73 | 94.67 | -0.43 | 101.85 | 88.31 | -1.63 | 108.17 | 1.05 |
| BMS-536924 | 56.86 | -3.49 | 40.57 | -4.84 | 48.71 | 86.04 | -1.95 | 93.73 | -0.80 |
| BEXAROTENE | 129.69 | 2.40 | 99.87 | -0.01 | 114.78 | 86.80 | -1.85 | 99.04 | -0.12 |
| CEDIRANIB | 69.90 | -2.43 | 57.22 | -3.48 | 63.56 | 86.10 | -1.94 | 80.88 | -2.45 |
| CANERTINIB | 28.59 | -5.77 | 34.33 | -5.34 | 31.46 | 84.09 | -2.22 | 50.65 | -6.32 |
| CI 1040 | 50.33 | -4.01 | 42.65 | -4.67 | 46.49 | 85.93 | -1.97 | 103.28 | 0.42 |
| CYCLOPAMINE | 141.65 | 3.37 | 104.03 | 0.33 | 122.84 | 85.59 | -2.01 | 106.32 | 0.81 |
| Lenalidomide | 144.91 | 3.63 | 144.60 | 3.63 | 144.76 | 84.86 | -2.12 | 104.96 | 0.63 |
| DASATINIB | 38.37 | -4.98 | 65.54 | -2.80 | 51.96 | 83.83 | -2.26 | 82.88 | -2.19 |
| PD 0325901 | 40.55 | -4.80 | 64.50 | -2.89 | 52.52 | 83.92 | -2.25 | 85.75 | -1.82 |
| 17-DMAG | 40.55 | -4.80 | 65.54 | -2.80 | 53.04 | 54.29 | -6.39 | 84.54 | -1.98 |
| PERIFOSINE | 86.21 | -1.11 | 89.47 | -0.86 | 87.84 | 83.07 | -2.37 | 103.68 | 0.47 |
| CHIR 258 | 66.64 | -2.70 | 66.58 | -2.72 | 66.61 | 81.15 | -2.64 | 93.31 | -0.86 |
| Etoposide | 77.51 | -1.82 | 72.82 | -2.21 | 75.17 | 88.49 | -1.61 | 103.75 | 0.48 |
| Allopurinol | 106.86 | 0.55 | 95.71 | -0.35 | 101.29 | 88.22 | -1.65 | 96.95 | -0.39 |
| Epirubicin hydrochloride | 44.90 | -4.45 | 45.77 | -4.41 | 45.34 | 86.21 | -1.93 | 76.16 | -3.05 |
| Thalidomide | 128.60 | 2.31 | 108.19 | 0.67 | 118.40 | 87.53 | -1.74 | 100.85 | 0.11 |
| Tretinoin | 117.73 | 1.43 | 106.11 | 0.50 | 111.92 | 87.25 | -1.78 | 80.43 | -2.51 |
| Bendamustine hydrochloride | 111.21 | 0.91 | 127.96 | 2.27 | 119.58 | 87.62 | -1.73 | 101.61 | 0.21 |
| Vemurafenib | 104.69 | 0.38 | 105.07 | 0.41 | 104.88 | 86.56 | -1.88 | 93.28 | -0.86 |
| Pentostatin | 134.04 | 2.75 | 146.68 | 3.80 | 140.36 | 86.56 | -1.88 | 97.96 | -0.26 |
| Vandetanib | 89.47 | -0.85 | 115.47 | 1.26 | 102.47 | 97.46 | -0.36 | 80.83 | -2.46 |
| Sorafenib | 5.76 | -7.62 | 15.60 | -6.87 | 10.68 | 71.32 | -4.01 | 88.55 | -1.47 |
| Abiraterone | 115.56 | 1.26 | 148.76 | 3.97 | 132.16 | 84.37 | -2.19 | 85.19 | -1.90 |
| Nilotinib | 126.43 | 2.14 | 113.39 | 1.09 | 119.91 | 85.20 | -2.07 | 94.65 | -0.68 |
| Temozolomide | 106.86 | 0.55 | 109.23 | 0.75 | 108.05 | 84.35 | -2.19 | 99.34 | -0.09 |
| Cladribine | 136.21 | 2.93 | 137.32 | 3.04 | 136.77 | 83.86 | -2.26 | 98.25 | -0.22 |
| Afatinib | 72.08 | -2.26 | 61.38 | -3.14 | 66.73 | 82.04 | -2.51 | 73.64 | -3.38 |
| Fulvestrant | 94.90 | -0.41 | 91.55 | -0.69 | 93.23 | 83.63 | -2.29 | 83.45 | -2.12 |
| Ponatinib | 53.59 | -3.75 | 58.26 | -3.40 | 55.93 | 80.95 | -2.66 | 84.93 | -1.93 |
| Daunorubicin hydrochloride | 48.16 | -4.19 | 61.38 | -3.14 | 54.77 | 81.24 | -2.62 | 65.12 | -4.47 |
| Cabazitaxel | 101.43 | 0.12 | 112.35 | 1.01 | 106.89 | 82.13 | -2.50 | 90.38 | -1.23 |
| Cisplatin | 89.47 | -0.85 | 98.83 | -0.10 | 94.15 | 82.43 | -2.46 | 99.37 | -0.08 |
| STF-62247 | 23.16 | -6.21 | 33.29 | -5.43 | 28.22 | 98.46 | -0.22 | 83.43 | -2.12 |
| GELDENAMYCIN | 70.99 | -2.34 | 86.35 | -1.11 | 78.67 | 94.70 | -0.74 | 102.35 | 0.30 |
| TRICHOSTATIN A | 147.09 | 3.81 | 94.67 | -0.43 | 120.88 | 97.32 | -0.37 | 33.58 | -8.51 |
| H-89 | 14.46 | -6.91 | 17.69 | -6.70 | 16.07 | 91.80 | -1.15 | 80.98 | -2.44 |
| Y-27632 | 76.42 | -1.91 | 74.90 | -2.04 | 75.66 | 96.21 | -0.53 | 95.76 | -0.54 |
| LAPATINIB | 118.82 | 1.52 | 95.71 | -0.35 | 107.26 | 85.57 | -2.02 | 87.41 | -1.61 |
| ELESCLOMOL | 30.77 | -5.60 | 35.37 | -5.26 | 33.07 | 74.49 | -3.57 | 99.01 | -0.13 |
| Lestaurtinib | 50.33 | -4.01 | 40.57 | -4.84 | 45.45 | 90.16 | -1.38 | 87.58 | -1.59 |
| ENZASTAURIN | 84.03 | -1.29 | 96.75 | -0.26 | 90.39 | 95.12 | -0.68 | 90.70 | -1.19 |
| MASITINIB | 5.76 | -7.62 | 16.64 | -6.78 | 11.20 | 86.99 | -1.82 | 52.92 | -6.03 |
| OBATOCLAX | 59.03 | -3.31 | 61.38 | -3.14 | 60.20 | 87.39 | -1.76 | 78.33 | -2.78 |
| BEZ 235 | 91.64 | -0.68 | 100.91 | 0.07 | 96.28 | 90.66 | -1.31 | 98.68 | -0.17 |
| AZD 2281 | 117.73 | 1.43 | 92.59 | -0.60 | 105.16 | 93.24 | -0.94 | 105.07 | 0.65 |
| NILOTINIB | 109.04 | 0.73 | 122.76 | 1.85 | 115.90 | 88.70 | -1.58 | 102.54 | 0.33 |
| SB 431542 | 112.30 | 0.99 | 114.43 | 1.17 | 113.37 | 90.72 | -1.30 | 93.14 | -0.88 |
| PD 98059 | 103.60 | 0.29 | 106.11 | 0.50 | 104.86 | 88.34 | -1.63 | 90.17 | -1.26 |
| PF-2341066 | 59.03 | -3.31 | 48.89 | -4.16 | 53.96 | 82.92 | -2.39 | 78.75 | -2.72 |
| PAZOPANIB | 100.34 | 0.03 | 122.76 | 1.85 | 111.55 | 86.91 | -1.83 | 97.40 | -0.33 |
| NVP AUY922 | 40.55 | -4.80 | 50.98 | -3.99 | 45.76 | 88.69 | -1.58 | 86.39 | -1.74 |
| PKC412 | 54.68 | -3.66 | 67.62 | -2.63 | 61.15 | 83.94 | -2.25 | 89.50 | -1.34 |
| Fludarabine phosphate | 112.30 | 0.99 | 119.64 | 1.60 | 115.97 | 95.37 | -0.65 | 119.94 | 2.55 |
| Celecoxib | 43.81 | -4.54 | 44.73 | -4.50 | 44.27 | 88.16 | -1.66 | 60.07 | -5.11 |
| Anastrozole | 89.47 | -0.85 | 99.87 | -0.01 | 94.67 | 95.38 | -0.65 | 100.06 | 0.01 |
| Imatinib | 60.12 | -3.22 | 52.02 | -3.90 | 56.07 | 94.04 | -0.83 | 90.17 | -1.26 |
| Letrozole | 99.25 | -0.06 | 115.47 | 1.26 | 107.36 | 93.68 | -0.88 | 99.90 | -0.01 |
| Pazopanib hydrochloride | 13.37 | -7.00 | 28.09 | -5.85 | 20.73 | 92.21 | -1.09 | 59.40 | -5.20 |
| Everolimus | 76.42 | -1.91 | 84.27 | -1.28 | 80.34 | 91.22 | -1.23 | 72.79 | -3.48 |
| Erlotinib hydrochloride | 118.82 | 1.52 | 73.86 | -2.13 | 96.34 | 93.40 | -0.92 | 82.34 | -2.26 |
| Sunitinib | 68.81 | -2.52 | 72.82 | -2.21 | 70.82 | 92.47 | -1.05 | 95.10 | -0.63 |
| Clofarabine | 146.00 | 3.72 | 131.08 | 2.53 | 138.54 | 94.15 | -0.82 | 100.89 | 0.11 |
| Regorafenib | 43.81 | -4.54 | 47.85 | -4.24 | 45.83 | 88.66 | -1.59 | 83.68 | -2.09 |
| 8-dodecylsulfanyl-1,3-dimethyl-7H-purine-2,6-dione | 112.30 | 0.99 | 118.60 | 1.51 | 115.45 | 93.89 | -0.85 | 103.16 | 0.40 |
| Oxaliplatin | 115.56 | 1.26 | 119.64 | 1.60 | 117.60 | 90.53 | -1.32 | 102.65 | 0.34 |
| Raloxifene | 56.86 | -3.49 | 52.02 | -3.90 | 54.44 | 90.90 | -1.27 | 91.29 | -1.12 |
| Vinorelbine tartrate | 231.88 | 10.66 | 185.18 | 6.93 | 208.53 | 88.42 | -1.62 | 110.51 | 1.35 |
| Lenalidomide | 107.95 | 0.64 | 129.00 | 2.36 | 118.47 | 92.38 | -1.07 | 92.77 | -0.93 |
| Zoledronic acid | 100.34 | 0.03 | 119.64 | 1.60 | 109.99 | 89.11 | -1.52 | 94.25 | -0.74 |
| Arsenic trioxide | 20.98 | -6.39 | 29.13 | -5.77 | 25.05 | 84.44 | -2.18 | 96.86 | -0.40 |
| Gefitinib | 97.08 | -0.24 | 102.99 | 0.24 | 100.03 | 87.95 | -1.68 | 85.80 | -1.82 |
| Floxuridine | 154.69 | 4.42 | 137.32 | 3.04 | 146.01 | 90.13 | -1.38 | 100.29 | 0.04 |
| HA14-1 | 80.77 | -1.55 | 97.79 | -0.18 | 89.28 | 102.67 | 0.37 | 99.78 | -0.03 |
| Roscovitine | 32.94 | -5.42 | 46.81 | -4.33 | 39.88 | 99.28 | -0.10 | 97.39 | -0.33 |
| SB 216763 | 88.38 | -0.94 | 106.11 | 0.50 | 97.25 | 101.41 | 0.20 | 100.07 | 0.01 |
| SB 202190 | 101.43 | 0.12 | 105.07 | 0.41 | 103.25 | 96.10 | -0.55 | 79.13 | -2.67 |
| MK-2206 | 48.16 | -4.19 | 47.85 | -4.24 | 48.01 | 95.56 | -0.62 | 85.95 | -1.80 |
| SB 203580 | 138.39 | 3.10 | 166.45 | 5.41 | 152.42 | 95.56 | -0.62 | 80.35 | -2.52 |
| PD 153035 | 143.82 | 3.54 | 121.72 | 1.77 | 132.77 | 98.57 | -0.20 | 83.75 | -2.08 |
| SORAFENIB | 4.67 | -7.70 | 7.28 | -7.54 | 5.98 | 80.39 | -2.74 | 91.34 | -1.11 |
| SU 11274 | 81.86 | -1.47 | 81.14 | -1.53 | 81.50 | 97.70 | -0.32 | 85.15 | -1.90 |
| SUNITINIB | 80.77 | -1.55 | 74.90 | -2.04 | 77.84 | 93.10 | -0.96 | 90.75 | -1.18 |
| Brivanib | 34.03 | -5.33 | 35.37 | -5.26 | 34.70 | 92.51 | -1.05 | 64.23 | -4.58 |
| Staurosporine | 23.16 | -6.21 | 14.56 | -6.95 | 18.86 | 82.83 | -2.40 | 101.58 | 0.20 |
| PXD101 | 105.78 | 0.47 | 106.11 | 0.50 | 105.94 | 96.16 | -0.54 | 87.87 | -1.55 |
| TANDUTINIB | 92.73 | -0.59 | 92.59 | -0.60 | 92.66 | 90.15 | -1.38 | 88.98 | -1.41 |
| BSI-201 | 102.51 | 0.20 | 112.35 | 1.01 | 107.43 | 93.94 | -0.85 | 101.73 | 0.22 |
| TEMSIROLIMUS | 86.21 | -1.11 | 83.22 | -1.36 | 84.72 | 87.05 | -1.81 | 65.73 | -4.39 |
| PCI-24781 | 118.82 | 1.52 | 98.83 | -0.10 | 108.83 | 93.46 | -0.91 | 91.15 | -1.13 |
| AG 490 | 118.82 | 1.52 | 120.68 | 1.68 | 119.75 | 89.42 | -1.48 | 85.62 | -1.84 |
| KU-55933 | 69.90 | -2.43 | 81.14 | -1.53 | 75.52 | 89.20 | -1.51 | 33.90 | -8.46 |
| VATALANIB | 64.47 | -2.87 | 69.70 | -2.47 | 67.08 | 87.19 | -1.79 | 78.45 | -2.76 |
| Cabozantinib | 27.50 | -5.86 | 27.05 | -5.94 | 27.28 | 93.87 | -0.86 | 67.75 | -4.13 |
| Vincristine sulfate | 200.35 | 8.11 | 196.62 | 7.86 | 198.49 | 98.56 | -0.20 | 109.71 | 1.24 |
| Pralatrexate | 117.73 | 1.43 | 106.11 | 0.50 | 111.92 | 96.60 | -0.47 | 103.37 | 0.43 |
| Cyclophosphamide | 107.95 | 0.64 | 117.56 | 1.43 | 112.75 | 98.87 | -0.16 | 101.62 | 0.21 |
| Omacetaxine mepesuccinate | 20.98 | -6.39 | 19.77 | -6.53 | 20.37 | 93.81 | -0.87 | 87.82 | -1.56 |
| Pipobroman | 125.34 | 2.05 | 121.72 | 1.77 | 123.53 | 98.07 | -0.27 | 102.03 | 0.26 |
| Pemetrexed | 112.30 | 0.99 | 110.27 | 0.84 | 111.29 | 94.67 | -0.74 | 94.26 | -0.73 |
| Streptozocin | 130.78 | 2.49 | 124.84 | 2.02 | 127.81 | 96.94 | -0.43 | 99.54 | -0.06 |
| Topotecan hydrochloride | 57.94 | -3.40 | 58.26 | -3.40 | 58.10 | 93.13 | -0.96 | 88.71 | -1.45 |
| Thioguanine | 93.82 | -0.50 | 110.27 | 0.84 | 102.05 | 96.19 | -0.53 | 92.95 | -0.90 |
| Dabrafenib mesylate | 126.43 | 2.14 | 121.72 | 1.77 | 124.07 | 93.50 | -0.91 | 100.50 | 0.06 |
| Dasatinib | 92.73 | -0.59 | 79.06 | -1.70 | 85.90 | 94.47 | -0.77 | 84.22 | -2.02 |
| Amifostine | 109.04 | 0.73 | 116.51 | 1.34 | 112.78 | 93.47 | -0.91 | 101.20 | 0.15 |
| Mechlorethamine hydrochloride | 107.95 | 0.64 | 117.56 | 1.43 | 112.75 | 76.70 | -3.26 | 100.62 | 0.08 |
| Exemestane | 109.04 | 0.73 | 96.75 | -0.26 | 102.89 | 91.81 | -1.14 | 90.38 | -1.23 |
| Dactinomycin | 60.12 | -3.22 | 65.54 | -2.80 | 62.83 | 93.25 | -0.94 | 95.88 | -0.53 |
| Enzalutamide | 94.90 | -0.41 | 98.83 | -0.10 | 96.87 | 90.81 | -1.28 | 80.08 | -2.55 |
| Uracil mustard | 76.42 | -1.91 | 72.82 | -2.21 | 74.62 | 93.89 | -0.85 | 97.98 | -0.26 |
| Nelarabine | 129.69 | 2.40 | 114.43 | 1.17 | 122.06 | 90.75 | -1.29 | 93.91 | -0.78 |
| Bortezomib | 89.47 | -0.85 | 117.56 | 1.43 | 103.51 | 93.30 | -0.94 | 83.42 | -2.12 |
| GSK1904529A | 116.65 | 1.35 | 92.59 | -0.60 | 104.62 | 100.76 | 0.11 | 86.97 | -1.67 |
| VANDETANIB | 85.12 | -1.20 | 88.43 | -0.94 | 86.77 | 85.66 | -2.01 | 102.19 | 0.28 |
| PF-04217903 | 90.56 | -0.76 | 78.02 | -1.79 | 84.29 | 99.86 | -0.02 | 111.09 | 1.42 |
| ZSTK474 | 95.99 | -0.32 | 74.90 | -2.04 | 85.45 | 95.63 | -0.61 | 86.44 | -1.74 |
| NVP LAQ824 | 93.82 | -0.50 | 83.22 | -1.36 | 88.52 | 98.78 | -0.17 | 95.25 | -0.61 |
| JNJ-26481585 | 112.30 | 0.99 | 109.23 | 0.75 | 110.77 | 96.89 | -0.43 | 97.58 | -0.31 |
| AG014699 | 65.55 | -2.78 | 67.62 | -2.63 | 66.59 | 96.59 | -0.48 | 82.53 | -2.24 |
| AZD1152-HQPA | 87.29 | -1.03 | 100.91 | 0.07 | 94.10 | 93.28 | -0.94 | 84.20 | -2.02 |
| PLX4720 | 30.77 | -5.60 | 42.65 | -4.67 | 36.71 | 93.90 | -0.85 | 79.36 | -2.64 |
| PLX4032 | 51.42 | -3.93 | 47.85 | -4.24 | 49.64 | 81.90 | -2.53 | 83.18 | -2.15 |
| METFORMIN | 117.73 | 1.43 | 114.43 | 1.17 | 116.08 | 91.06 | -1.25 | 96.22 | -0.48 |
| 2DG | 104.69 | 0.38 | 130.04 | 2.44 | 117.36 | 93.82 | -0.86 | 91.76 | -1.06 |
| Vorinostat | 88.38 | -0.94 | 88.43 | -0.94 | 88.40 | 101.32 | 0.19 | 97.95 | -0.26 |
| Bosutinib | 10.11 | -7.26 | 17.69 | -6.70 | 13.90 | 75.29 | -3.45 | 70.20 | -3.82 |
| Irinotecan hydrochloride | 116.65 | 1.35 | 122.76 | 1.85 | 119.70 | 97.13 | -0.40 | 96.04 | -0.51 |
| Temsirolimus | 50.33 | -4.01 | 62.42 | -3.06 | 56.38 | 93.12 | -0.96 | 68.61 | -4.02 |
| Doxorubicin hydrochloride | 49.25 | -4.10 | 58.26 | -3.40 | 53.75 | 94.10 | -0.82 | 78.74 | -2.72 |
| Mitoxantrone | 5.76 | -7.62 | 15.60 | -6.87 | 10.68 | 90.67 | -1.30 | 40.29 | -7.65 |
| Estramustine phosphate sodium | 26.42 | -5.95 | 38.49 | -5.00 | 32.45 | 92.97 | -0.98 | 86.35 | -1.75 |
| Vismodegib | 175.35 | 6.09 | 147.72 | 3.88 | 161.54 | 93.24 | -0.95 | 90.92 | -1.16 |
| Axitinib | 32.94 | -5.42 | 31.21 | -5.60 | 32.07 | 91.66 | -1.17 | 24.49 | -9.67 |
| Trametinib | 101.43 | 0.12 | 79.06 | -1.70 | 90.25 | 92.79 | -1.01 | 95.81 | -0.54 |
| Plerixafor | 112.30 | 0.99 | 111.31 | 0.92 | 111.81 | 92.88 | -1.00 | 97.43 | -0.33 |
| Bleomycin sulfate | 125.34 | 2.05 | 143.56 | 3.54 | 134.45 | 80.17 | -2.77 | 94.27 | -0.73 |
| BMS 204352 | 10.11 | -7.26 | 17.69 | -6.70 | 13.90 | 103.30 | 0.46 | 36.55 | -8.12 |
| FK-506 | 80.77 | -1.55 | 91.55 | -0.69 | 86.16 | 113.03 | 1.82 | 77.49 | -2.88 |
| CHIR 98014 | 60.12 | -3.22 | 53.06 | -3.82 | 56.59 | 110.23 | 1.43 | 78.77 | -2.72 |
| RAPAMYCIN | 95.99 | -0.32 | 74.90 | -2.04 | 85.45 | 110.83 | 1.51 | 77.09 | -2.93 |
| BX 912 | 35.11 | -5.24 | 40.57 | -4.84 | 37.84 | 106.22 | 0.87 | 85.82 | -1.82 |
| SARACATINIB | 85.12 | -1.20 | 98.83 | -0.10 | 91.97 | 108.09 | 1.13 | 107.97 | 1.02 |
| GW 843682X | 163.39 | 5.12 | 170.61 | 5.75 | 167.00 | 106.44 | 0.90 | 102.89 | 0.37 |
| AZD 6244 | 100.34 | 0.03 | 90.51 | -0.77 | 95.42 | 108.13 | 1.14 | 96.05 | -0.51 |
| SU 6656 | 128.60 | 2.31 | 106.11 | 0.50 | 117.36 | 105.95 | 0.83 | 101.36 | 0.17 |
| ABT-737 | 132.95 | 2.66 | 126.92 | 2.19 | 129.94 | 107.35 | 1.03 | 95.47 | -0.58 |
| PD 180970 | 120.99 | 1.70 | 116.51 | 1.34 | 118.75 | 102.30 | 0.32 | 100.96 | 0.12 |
| ABT-888 | 75.34 | -1.99 | 93.63 | -0.52 | 84.48 | 107.03 | 0.98 | 105.22 | 0.67 |
| GSK 269962A | 26.42 | -5.95 | 31.21 | -5.60 | 28.81 | 101.41 | 0.20 | 79.80 | -2.59 |
| BI 2536 | 40.55 | -4.80 | 46.81 | -4.33 | 43.68 | 99.22 | -0.11 | 108.74 | 1.12 |
| U 73122 | 128.60 | 2.31 | 129.00 | 2.36 | 128.80 | 102.60 | 0.36 | 109.98 | 1.28 |
| BMS-599626 | 38.37 | -4.98 | 50.98 | -3.99 | 44.68 | 101.94 | 0.27 | 83.05 | -2.17 |
| DEGUELIN | 27.50 | -5.86 | 26.01 | -6.02 | 26.76 | 100.58 | 0.08 | 66.30 | -4.32 |
| BIBW2992 | 70.99 | -2.34 | 94.67 | -0.43 | 82.83 | 100.79 | 0.11 | 76.41 | -3.02 |
| LE 135 | 44.90 | -4.45 | 74.90 | -2.04 | 59.90 | 98.37 | -0.23 | 97.72 | -0.29 |
| ERLOTINIB | 90.56 | -0.76 | 81.14 | -1.53 | 85.85 | 103.32 | 0.46 | 80.96 | -2.44 |
| Tamoxifen citrate | 4.67 | -7.70 | 1.04 | -8.05 | 2.86 | 43.38 | -7.92 | 59.05 | -5.24 |
| Carboplatin | 110.12 | 0.82 | 114.43 | 1.17 | 112.28 | 109.23 | 1.29 | 96.43 | -0.46 |
| Crizotinib | 35.11 | -5.24 | 34.33 | -5.34 | 34.72 | 101.94 | 0.27 | 91.93 | -1.03 |
| Carfilzomib | 91.64 | -0.68 | 80.10 | -1.62 | 85.87 | 107.54 | 1.05 | 97.18 | -0.36 |
| Docetaxel | 174.26 | 6.00 | 155.01 | 4.48 | 164.63 | 107.04 | 0.98 | 101.54 | 0.20 |
| Teniposide | 119.91 | 1.61 | 125.88 | 2.11 | 122.89 | 104.70 | 0.66 | 92.82 | -0.92 |
| Decitabine | 185.13 | 6.88 | 197.66 | 7.95 | 191.40 | 106.21 | 0.87 | 110.67 | 1.37 |
| Sirolimus | 115.56 | 1.26 | 105.07 | 0.41 | 110.32 | 102.71 | 0.38 | 80.58 | -2.49 |
| Valrubicin | 135.13 | 2.84 | 114.43 | 1.17 | 124.78 | 104.29 | 0.60 | 92.36 | -0.98 |
| Lomustine | 101.43 | 0.12 | 131.08 | 2.53 | 116.25 | 102.96 | 0.41 | 109.33 | 1.20 |
| NSC 625987 | 85.12 | -1.20 | 85.31 | -1.20 | 85.21 | 109.17 | 1.28 | 78.37 | -2.77 |
| Fingolimod | 99.25 | -0.06 | 107.15 | 0.58 | 103.20 | 110.17 | 1.42 | 94.97 | -0.64 |
| GW 441756 | 93.82 | -0.50 | 71.78 | -2.30 | 82.80 | 107.67 | 1.07 | 109.93 | 1.27 |
| GEFITINIB | 116.65 | 1.35 | 127.96 | 2.27 | 122.30 | 108.39 | 1.17 | 97.47 | -0.32 |
| DR 2313 | 86.21 | -1.11 | 96.75 | -0.26 | 91.48 | 106.28 | 0.88 | 93.50 | -0.83 |
| GDC-0449 | 138.39 | 3.10 | 124.84 | 2.02 | 131.61 | 106.81 | 0.95 | 95.45 | -0.58 |
| PD 166793 | 104.69 | 0.38 | 86.35 | -1.11 | 95.52 | 105.53 | 0.77 | 99.99 | 0.00 |
| HYPOTHEMYCIN | 53.59 | -3.75 | 48.89 | -4.16 | 51.24 | 103.00 | 0.42 | 72.32 | -3.54 |
| AEG 3482 | 122.08 | 1.78 | 113.39 | 1.09 | 117.74 | 105.28 | 0.74 | 93.96 | -0.77 |
| IMATINIB | 73.16 | -2.17 | 92.59 | -0.60 | 82.87 | 103.64 | 0.51 | 81.63 | -2.35 |
| PIK 75 | 23.16 | -6.21 | 53.06 | -3.82 | 38.11 | 101.60 | 0.22 | 87.68 | -1.58 |
| LBH-589 | 75.34 | -1.99 | 97.79 | -0.18 | 86.56 | 105.58 | 0.78 | 101.54 | 0.20 |
| SD 169 | 126.43 | 2.14 | 116.51 | 1.34 | 121.47 | 104.03 | 0.56 | 109.31 | 1.19 |
| MGCD0103 | 77.51 | -1.82 | 75.94 | -1.96 | 76.73 | 103.72 | 0.52 | 90.12 | -1.27 |
| PIK 90 | 98.17 | -0.15 | 86.35 | -1.11 | 92.26 | 101.53 | 0.21 | 102.65 | 0.34 |
| MS-275 | 63.38 | -2.96 | 70.74 | -2.38 | 67.06 | 101.17 | 0.16 | 107.21 | 0.92 |
| PD 169316 | 109.04 | 0.73 | 107.15 | 0.58 | 108.09 | 100.95 | 0.13 | 97.78 | -0.28 |
| MOTESANIB | 78.05 | -1.77 | 89.47 | -0.86 | 83.76 | 99.74 | -0.04 | 76.36 | -3.03 |
| LY 294002 | 88.38 | -0.94 | 76.98 | -1.87 | 82.68 | 99.01 | -0.14 | 80.46 | -2.50 |
| Cytarabine hydrochloride | 93.82 | -0.50 | 101.95 | 0.16 | 97.88 | 107.99 | 1.12 | 93.77 | -0.80 |
| Thiotepa | 104.69 | 0.38 | 125.88 | 2.11 | 115.28 | 107.20 | 1.01 | 95.81 | -0.54 |
| Melphalan hydrochloride | 79.68 | -1.64 | 98.83 | -0.10 | 89.26 | 107.21 | 1.01 | 85.47 | -1.86 |
| Dacarbazine | 137.30 | 3.01 | 133.16 | 2.70 | 135.23 | 105.89 | 0.82 | 99.29 | -0.09 |
| Chlorambucil | 262.32 | 13.12 | 243.43 | 11.67 | 252.88 | 105.04 | 0.71 | 97.90 | -0.27 |
| Megestrol acetate | 152.52 | 4.24 | 135.24 | 2.87 | 143.88 | 105.65 | 0.79 | 99.64 | -0.05 |
| Triethylenemelamine | 118.82 | 1.52 | 116.51 | 1.34 | 117.67 | 105.86 | 0.82 | 108.47 | 1.08 |
| Azacitidine | 95.99 | -0.32 | 102.99 | 0.24 | 99.49 | 102.96 | 0.41 | 93.68 | -0.81 |
| Altretamine | 92.73 | -0.59 | 122.76 | 1.85 | 107.74 | 103.08 | 0.43 | 97.84 | -0.28 |
| Aminolevulinic acid hydrochloride | 99.25 | -0.06 | 109.23 | 0.75 | 104.24 | 102.19 | 0.31 | 100.02 | 0.00 |
| NU 1025 | 95.99 | -0.32 | 46.81 | -4.33 | 71.40 | 111.16 | 1.56 | 89.72 | -1.32 |
| RDEA119 | 12.28 | -7.09 | 9.36 | -7.37 | 10.82 | 102.46 | 0.34 | 66.81 | -4.25 |
| IVACHTIN | 112.30 | 0.99 | 60.34 | -3.23 | 86.32 | 108.95 | 1.25 | 102.63 | 0.34 |
| ROFECOXIB | 122.08 | 1.78 | 53.06 | -3.82 | 87.57 | 109.17 | 1.28 | 102.66 | 0.34 |
| CHIR 99021 | 38.37 | -4.98 | 31.21 | -5.60 | 34.79 | 107.66 | 1.07 | 70.15 | -3.82 |
| VORINOSTAT | 105.78 | 0.47 | 75.94 | -1.96 | 90.86 | 108.87 | 1.24 | 84.44 | -1.99 |
| SD 208 | 136.21 | 2.93 | 54.10 | -3.73 | 95.16 | 107.60 | 1.06 | 88.62 | -1.46 |
| TOZASERTIB | 80.77 | -1.55 | 26.01 | -6.02 | 53.39 | 106.13 | 0.86 | 73.72 | -3.37 |
| BX 795 | 47.07 | -4.28 | 28.09 | -5.85 | 37.58 | 103.33 | 0.47 | 70.68 | -3.76 |
| VX-702 | 116.65 | 1.35 | 67.62 | -2.63 | 92.13 | 105.35 | 0.75 | 93.91 | -0.78 |
| AZD 7762 | 82.95 | -1.38 | 67.62 | -2.63 | 75.28 | 102.48 | 0.35 | 92.74 | -0.93 |
| ZM447439 | 120.99 | 1.70 | 79.06 | -1.70 | 100.03 | 106.45 | 0.90 | 98.24 | -0.23 |
| LY 333531 | 41.64 | -4.72 | 27.05 | -5.94 | 34.34 | 101.02 | 0.14 | 78.46 | -2.76 |
| NVP TAE684 | 9.02 | -7.35 | 5.20 | -7.71 | 7.11 | 37.86 | -8.69 | 96.39 | -0.46 |
| AS 252424 | 59.03 | -3.31 | 40.57 | -4.84 | 49.80 | 99.49 | -0.07 | 69.90 | -3.86 |
| Methoxsalen | 106.86 | 0.55 | 112.35 | 1.01 | 109.61 | 108.78 | 1.23 | 93.90 | -0.78 |
| Procarbazine hydrochloride | 118.82 | 1.52 | 96.75 | -0.26 | 107.78 | 108.68 | 1.21 | 99.34 | -0.08 |
| Vinblastine sulfate | 217.75 | 9.52 | 165.41 | 5.32 | 191.58 | 107.53 | 1.05 | 109.75 | 1.25 |
| Imiquimod | 116.65 | 1.35 | 112.35 | 1.01 | 114.50 | 107.35 | 1.03 | 104.72 | 0.60 |
| Busulfan | 93.82 | -0.50 | 112.35 | 1.01 | 103.09 | 106.60 | 0.92 | 97.98 | -0.26 |
| Ifosfamide | 115.56 | 1.26 | 99.87 | -0.01 | 107.71 | 105.83 | 0.82 | 96.59 | -0.44 |
| Capecitabine | 124.26 | 1.96 | 114.43 | 1.17 | 119.35 | 107.18 | 1.00 | 100.06 | 0.01 |
| Mercaptopurine | 118.82 | 1.52 | 112.35 | 1.01 | 115.59 | 106.22 | 0.87 | 102.01 | 0.26 |
| Fluorouracil | 107.95 | 0.64 | 116.51 | 1.34 | 112.23 | 104.34 | 0.61 | 111.63 | 1.49 |
| Methotrexate | 109.04 | 0.73 | 102.99 | 0.24 | 106.01 | 101.95 | 0.27 | 94.27 | -0.73 |
| Triciribine | 136.21 | 2.93 | 138.36 | 3.12 | 137.29 | 115.58 | 2.18 | 106.29 | 0.80 |
| PI-103 | 111.21 | 0.91 | 94.67 | -0.43 | 102.94 | 118.92 | 2.64 | 106.19 | 0.79 |
| CHK2 INHIBITOR II | 26.42 | -5.95 | 20.81 | -6.44 | 23.61 | 112.91 | 1.80 | 68.27 | -4.06 |
| AS-604850 | 65.55 | -2.78 | 75.94 | -1.96 | 70.75 | 116.10 | 2.25 | 62.77 | -4.77 |
| COMPOUND 401 | 98.17 | -0.15 | 94.67 | -0.43 | 96.42 | 115.81 | 2.21 | 93.50 | -0.83 |
| TGX 221 | 110.12 | 0.82 | 123.80 | 1.94 | 116.96 | 115.69 | 2.19 | 101.89 | 0.24 |
| DNA-PK INHIBITOR V | 92.73 | -0.59 | 99.87 | -0.01 | 96.30 | 115.08 | 2.11 | 82.55 | -2.23 |
| COMPOUND C | 137.30 | 3.01 | 115.47 | 1.26 | 126.39 | 115.42 | 2.16 | 104.57 | 0.59 |
| R59022 | 76.42 | -1.91 | 98.83 | -0.10 | 87.63 | 113.72 | 1.92 | 55.65 | -5.68 |
| AS-041164 | 82.95 | -1.38 | 106.11 | 0.50 | 94.53 | 113.38 | 1.87 | 84.77 | -1.95 |
| JNK INHIBITOR X | 1.41 | -7.97 | 3.12 | -7.88 | 2.27 | 82.79 | -2.41 | 4.80 | -12.19 |
| GDC 0941 | 115.56 | 1.26 | 94.67 | -0.43 | 105.11 | 113.65 | 1.91 | 84.36 | -2.00 |
| NSC23766 | 101.43 | 0.12 | 99.87 | -0.01 | 100.65 | 111.37 | 1.59 | 106.21 | 0.79 |
| Pp121 | 39.46 | -4.89 | 58.26 | -3.40 | 48.86 | 111.23 | 1.57 | 124.27 | 3.11 |
| SB 218078 | 80.77 | -1.55 | 78.02 | -1.79 | 79.40 | 109.07 | 1.27 | 92.40 | -0.97 |
| SP600125 | 112.30 | 0.99 | 85.31 | -1.20 | 98.80 | 108.63 | 1.21 | 110.92 | 1.40 |
| A-769662 | 118.82 | 1.52 | 112.35 | 1.01 | 115.59 | 107.48 | 1.05 | 79.85 | -2.58 |
| AICAR | 100.34 | 0.03 | 110.27 | 0.84 | 105.31 | 108.29 | 1.16 | 107.04 | 0.90 |
| Mitomycin | 109.04 | 0.73 | 99.87 | -0.01 | 104.45 | 117.51 | 2.45 | 110.98 | 1.41 |
| Lapatinib | 116.65 | 1.35 | 76.98 | -1.87 | 96.81 | 114.10 | 1.97 | 75.39 | -3.15 |
| Gemcitabine hydrochloride | 103.60 | 0.29 | 94.67 | -0.43 | 99.13 | 117.16 | 2.40 | 102.42 | 0.31 |
| Carmustine | 106.86 | 0.55 | 102.99 | 0.24 | 104.93 | 117.12 | 2.39 | 102.90 | 0.37 |
| Plicamycin | 85.12 | -1.20 | 86.35 | -1.11 | 85.73 | 115.83 | 2.21 | 92.59 | -0.95 |
| Mitotane | 24.24 | -6.12 | 36.41 | -5.17 | 30.33 | 93.94 | -0.85 | 54.78 | -5.79 |
| Hydroxyurea | 89.47 | -0.85 | 112.35 | 1.01 | 100.91 | 115.47 | 2.16 | 102.60 | 0.33 |
| Dexrazoxane | 115.56 | 1.26 | 118.60 | 1.51 | 117.08 | 114.95 | 2.09 | 109.07 | 1.16 |
| Idarubicin hydrochloride | 101.43 | 0.12 | 91.55 | -0.69 | 96.49 | 112.79 | 1.79 | 114.41 | 1.85 |
| Paclitaxel | 87.29 | -1.03 | 94.67 | -0.43 | 90.98 | 111.36 | 1.59 | 114.27 | 1.83 |
